# Variations in the prevalence of scoliosis by age, sex, geographic region, and subtype among Chinese children: A systematic review and modelling study

**DOI:** 10.7189/jogh.14.04062

**Published:** 2024-04-12

**Authors:** Jin Cao, Weidi Sun, Yutong Zheng, Shiyi Shan, Yi Liu, Xuanyin Huang, Ke Tang, Yajie Zhu, Davies Adeloye, Igor Rudan, Peige Song, Global Health Epidemiology Research Group (GHERG)

**Affiliations:** 1 School of Public Health and the Second Affiliated Hospital, Zhejiang University School of Medicine, Zhejiang University, Hangzhou, Zhejiang, China; 2 School of Public Health, Zhejiang University School of Medicine, Zhejiang University, Hangzhou, Zhejiang, China; 3 School of Information Science and Technology, Hangzhou Normal University, Hangzhou, China; 4 Centre for Global Health, Usher Institute of Population Health Sciences and Informatics, University of Edinburgh, Edinburgh, Scotland, UK; 5 Algebra University, Zagreb, Croatia

## Abstract

**Background:**

Scoliosis is a prevalent spinal condition typically detected in children, yet its prevalence in China has not been fully understood. This study aimed to estimate the prevalence of scoliosis among Chinese children and explore its variations by age, sex, geographic region, and subtype.

**Methods:**

We conducted a comprehensive search of seven bibliographic databases to identify epidemiological studies that reported the prevalence of scoliosis among Chinese children published since 1990. Scoliosis was defined as a Cobb angle equal to or greater than 10°, as detected radiographically. We used a multilevel mixed-effect meta-regression to estimate the age- and sex-specific prevalence of idiopathic scoliosis (IS). The overall prevalence and total cases of IS among Chinese children aged 5–18 years in 2020 were generated by applying the China census data. A random-effects meta-analysis was conducted to estimate the pooled prevalence of IS with varying curvatures (10–19°, 20–39°, and ≥40°) and locations (thoracic curve, lumbar curve, thoracolumbar curve, and double curve), as well as congenital scoliosis (CS).

**Results:**

A total of 46 articles covering 1 112 163 Chinese children were included. The prevalence of IS ranged from 0.06% (95% confidence interval (CI) = 0.00–0.26) among children aged five years to 1.44% (95% CI = 0.89–2.13) among those aged 18 years, peaking at 1.79% (95% CI = 1.18–2.53) among those aged 16 years. In 2020, the overall prevalence of IS among Chinese children aged 5–18 was 0.79% (95% CI = 0.45–1.27), translating to an estimated 1.81 million (95% CI = 1.04–2.91) affected children. Notably, IS prevalence was nearly double in girls compared to boys (1.03%, 95% CI = 0.63–1.57 vs. 0.58%, 95% CI = 0.29–1.01). Among the six geographic regions, the prevalence of IS was the highest in Northwest China (1.54%, 95% CI = 0.75–2.65) and the lowest in North China (0.33%, 95% CI = 0.11–0.73). The most commonly identified IS subtypes were curvatures of 10–19° and thoracolumbar curves, with a pooled prevalence of 7.14‰ (95% CI = 3.44–12.15) and 2.00‰ (95% CI = 0.83–3.65), respectively. As for CS, the pooled prevalence was 3.03 per 10 000 (95% CI = 1.88–4.43).

**Conclusions:**

The prevalence of scoliosis among Chinese children exhibits considerable variations across age, sex, geographic regions, and subtypes. It is imperative to develop targeted public health strategies and optimise the allocation of regional health resources. High-quality epidemiological research is still needed to unmask the diverse burden of scoliosis in children.

**Registration:**

PROSPERO CRD42023406260.

Scoliosis is a common spinal condition, usually diagnosed in children and adolescents [1]. It is characterised by a three-dimensional deviation of the spine, which is diagnosed by measuring the Cobb angle on posterior-anterior radiographs. A Cobb angle ≥10° indicates the presence of scoliosis [1]. The condition is typically classified into three primary subtypes: idiopathic scoliosis (IS), congenital scoliosis (CS), and neuromuscular scoliosis (NS) [2]. IS, characterised by an unknown cause, is the most prevalent form and accounts for approximately 80% of all diagnosed scoliosis cases [3]. CS arises from embryological or intrauterine vertebral maldevelopment [1], while NS is associated with neuromuscular conditions such as cerebral palsy or muscular dystrophy [4].

Scoliosis in children often manifests prior to musculoskeletal maturity, leading to significant physical and psychological health problems, such as back pain, impaired functional capacity, depression, and anxiety [5–7]. The early stages of scoliosis are typically asymptomatic, which can result in delayed recognition and potential exacerbation of the condition’s progression [8]. Neglecting the early signs of scoliosis can increase the risk of severe spinal deformities, profound cardiopulmonary compromise, respiratory dysfunction, and even disability [9]. Notably, when the scoliosis angle exceeds 30° during childhood, it significantly raises the risk of developing degenerative scoliosis in adulthood – a condition associated with various health problems and a diminished quality of life [10–12]. Therefore, it is crucial to prioritise reliable screening and develop a comprehensive understanding of the epidemiological characteristics of scoliosis in the paediatric population. These efforts can improve the health outcomes for individuals affected by this condition and, by extension, benefit the overall health of the population. Globally, the prevalence of scoliosis varies from 0.11–5.2%, which can be attributed to diverse population characteristics, geographical influences, and etiological factors [8,13–17]. Adolescence and female sex have been identified as important risk factors for scoliosis [18–21]. Additionally, some environmental factors, such as latitude, have been suggested as potential contributors to scoliosis, although further validation is required to explore their association [22,23].

China is one of the most populous countries in the world, with a significant proportion of its population consisting of children aged 0–18 years [24,25]. However, due to the expansive geographical scale and disparate levels of economic development across China, the distribution of diseases demonstrates substantial intra-national variations. Previous epidemiological investigations have unveiled a significant disparity in the prevalence of scoliosis among Chinese children, ranging from 0.11% in Beijing to 2.40% in Sichuan [26,27]. A systematic review and meta-analysis published in 2015, based on 38 population-based articles, reported a pooled prevalence of scoliosis among Chinese children at 1.02%, with a female-to-male ratio of 1.54 [28]. By far, no recent study has systematically assessed the prevalence of scoliosis among Chinese children, let alone its variations by age, sex, region, and subtype.

To fill this gap in knowledge, we conducted a comprehensive review of the literature to synthesise the best epidemiological evidence since 1990. We aimed to estimate the prevalence of scoliosis among Chinese children, with a focus on its variations in age, sex, geographic region, and subtype.

## METHODS

This systematic review and modelling study was registered on PROSPERO CRD42023406260. The review followed the Preferred Reporting Items for Systematic Reviews and Meta-Analyses (PRISMA) guidelines and adhered to the Guidelines for Accurate and Transparent Health Estimates Reporting (GATHER) statement [29,30].

### Search strategy

To identify all published studies on the prevalence of scoliosis in Chinese children, a comprehensive search strategy was conducted across four Chinese and three English bibliographic databases: China National Knowledge Infrastructure (CNKI), Wanfang, Chinese Biomedicine Literature Database (CBM), CQVIP, PubMed, EMBASE, and Medline. The search was limited to studies published between January 1990 and June 2023. The search strategy combined medical subject headings terms and free text terms related to scoliosis, epidemiology (prevalence, incidence, epidemiology, morbidity), children (adolescent, infant, boys, girls, child, student), and China (China, Chinese, Hong Kong, Macao, Taiwan; only in English databases). The specific search strategies for each bibliographic database are described in Table S1 in the **Online Supplementary Document**. No language restrictions were applied during the search or selection process. Additionally, the reference lists of all included studies were also manually searched to identify potentially relevant studies.

### Selection criteria

This review included studies that reported the prevalence of scoliosis among the general Chinese paediatric population. Scoliosis should have been defined as a Cobb angle greater than or equal to 10° and diagnosed through radiographs by a doctor. Studies that focused on specific paediatric populations (e.g. obese children and those who had undergone lower extremity surgery in the past) were excluded due to their poor representation of the general paediatric population. To analyse the prevalence of scoliosis subtypes, we excluded studies that only provided the prevalence of overall scoliosis without specifying its subtypes. Additionally, abstracts, letters, reviews, viewpoints, or case reports were also excluded. For articles with an overlapping study population, only the most representative one (most recent, largest sample, and most comprehensive description) was included for further analysis.

### Data extraction

Two researchers (JC and WS) independently screened all records in two stages (title and abstract screening and full-text review) after eliminating all duplicate citations. Scoliosis was classified into three subtypes: IS, CS, and NS. A pilot-tested and refined data extraction form was used to extract relevant data from the included articles.

Two researchers (JC and YZ) independently extracted information on study characteristics (authors, publication year, study setting, study design, investigation date, sampling strategy), population characteristics (girl proportion, age range, or average age), diagnostic criteria for scoliosis, examination methods, and prevalence data (sample size and number of children with scoliosis by age group, sex, scoliosis subtype, curvature and location, where available).

To classify studies into different geographic regions, we used the definitions of the National Bureau of Statistics of China. The six identified regions were East China, North China, Northeast China, Northwest China, South Central China, and Southwest China (Figure S1 in the **Online Supplementary Document**) [24]. The latitude and longitude of the investigation sites were obtained by Global Positioning System coordinates from Google Maps (http://www.gps-coordinates.net/). Any discrepancies in the study selection and data extraction process were resolved by consensus with the help of a senior reviewer (PS).

### Quality assessment

Two reviewers (WS and YZ) assessed the quality of each included article using a modified version of a critical appraisal tool developed for prevalence systematic reviews [31]. The appraisal tool consisted of nine items, and a score of one (yes) or zero (no) was assigned for each item based on the assessment. The overall quality score for each article ranged from zero to nine (Table S2 in the **Online Supplementary Document**).

### Statistical analysis

To enable the inclusion of zero prevalence reported in some specific subgroups, a value of 0.0005 was adopted to replace zero cells. The variance of prevalence was stabilised using the Freeman-Tukey double arcsine transformation [32].

#### Meta-regression of the prevalence of IS

For IS, one individual article may have contributed multiple data points for different age groups, sexes, or settings. To account for this hierarchical data structure, a multilevel mixed-effect meta-regression model was adopted. The nonlinear association of average age and IS prevalence was modelled using a restricted cubic spline. Before constructing the models for IS prevalence, a univariable meta-regression analysis was first used to explore the associations between IS prevalence and cluster-level variables, including average age, girl proportion, geographic region, geographic indicators (latitude and longitude), publication year and study year (Table S3 in the **Online Supplementary Document**). Average age, girl proportion, and geographic region were found to be significantly associated with the IS prevalence and, therefore, were included in the subsequent multilevel mixed-effect meta-regression analysis. The age range was set as 5–18 years, where most data points were concentrated (Figure S2 in the **Online Supplementary Document**). Additionally, we included interaction terms of girl proportion and average age. Finally, the age- and sex-specific prevalence of IS in different geographic regions of China were generated based on the final model. The details of the modelling process can be found in Table S4 in the **Online Supplementary Document**.

#### Estimation of the national and regional number of children with IS in 2020

The number of children with IS in the six geographic regions was respectively calculated by multiplying the age- and sex-specific IS prevalence estimates with the corresponding paediatric population data obtained from the seventh national census of Mainland China [24]. The national number of children with IS in China was then generated by summing the number of children with IS in the six geographic regions.

#### Meta-analysis of IS prevalence by curvature and location and CS prevalence

A random-effects meta-analysis was used to estimate the pooled prevalence of IS with different curvatures (10–19°, 20–39°, and ≥40°) and locations (thoracic curve, lumbar curve, thoracolumbar curve, and double curve), as well as CS. Heterogeneity between studies was assessed using Cochran’s Q statistic (*P* < 0.05 indicated significant heterogeneity) and the *I^2^* statistic (≥50% indicated substantial heterogeneity) [33,34].

Publication bias was visually evaluated with funnel plots or tested by Egger’s test and Begg’s test (*P* < 0.05 indicated significant publication bias) when the number of included articles was 10 or more [35,36]. When publication bias was detected, we used the trim-and-fill method to adjust the effect estimate [37]. Additionally, we conducted a leave-one-out sensitivity analysis to assess the robustness of our results. All analysis was conducted using *R*, version 4.3.2 (R Foundation for Statistical Computing, Vienna, Austria).

## RESULTS

### Study selection and characteristics

**Figure 1:**
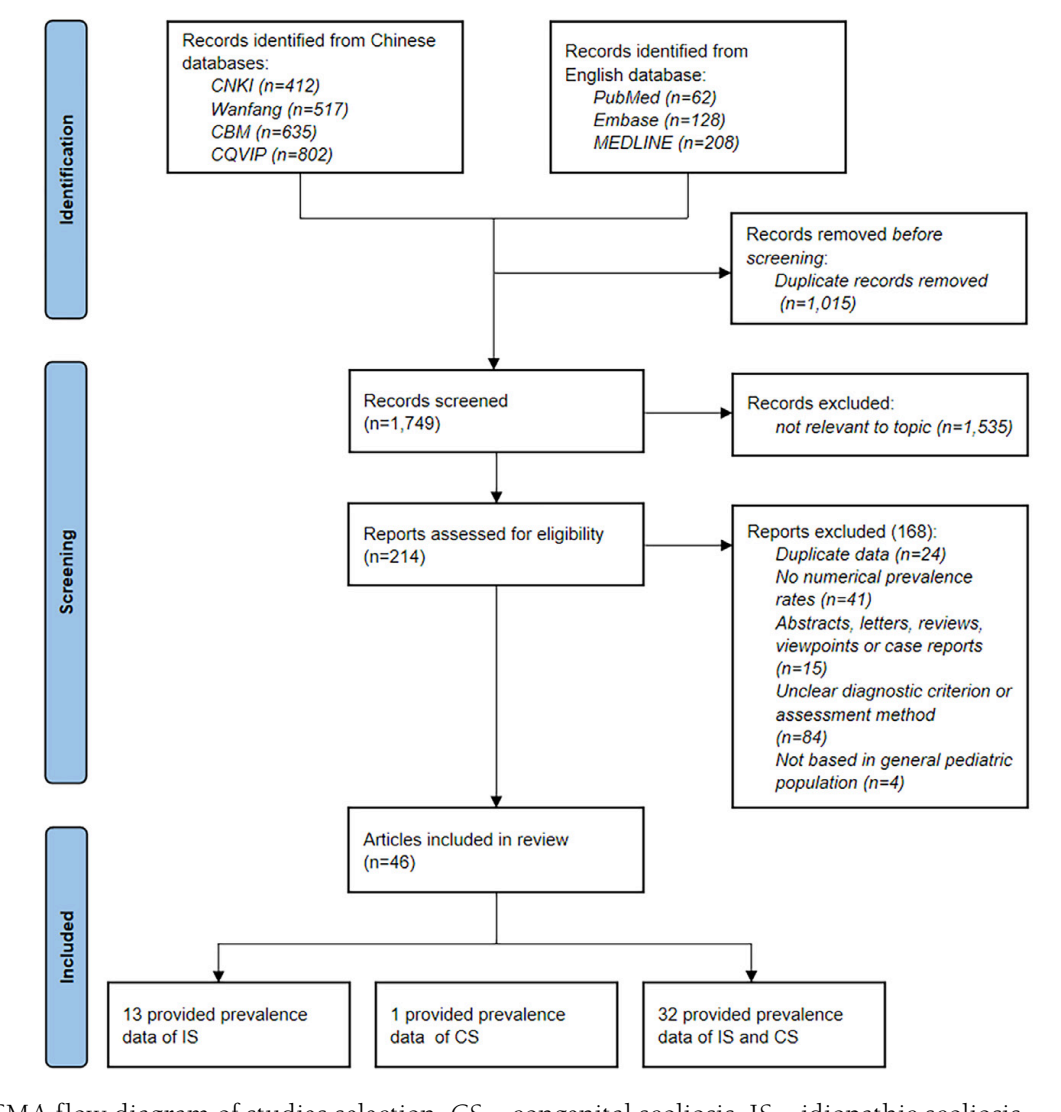
PRISMA flow diagram of studies selection. CS – congenital scoliosis, IS – idiopathic scoliosis

A total of 2764 records were initially retrieved from the database research. After removing 1015 duplicates, 1749 records remained for the title and abstract screening. Subsequently, 214 articles were reviewed for full-text. Finally, 46 articles covering a total of 1 112 163 children were deemed eligible for this systematic review. Among these articles, 13 reported the prevalence of IS, one reported the prevalence of CS, and 32 reported the prevalence of both IS and CS (**Figure 1**).

**Figure 2:**
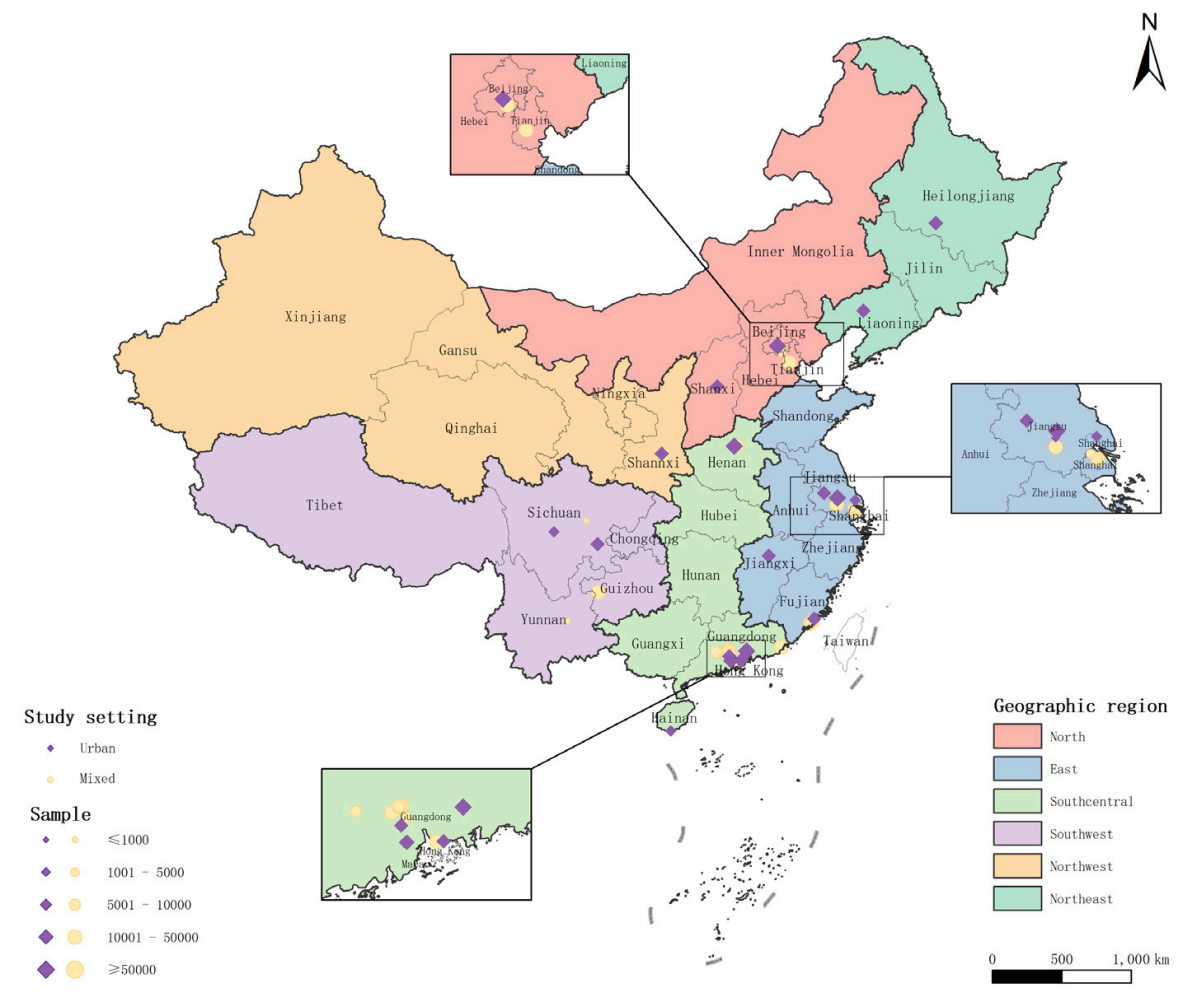
Geographic distribution of the included articles (n = 46).

Of all the 46 included articles, 43 (93.48%) were assigned an overall quality score of five points or higher (Table S2 in the **Online Supplementary Document**). **Table 1** presents a summary of the main characteristics of all 46 included articles. The majority (n = 30, 65.22%) were published between 2010 and 2019. Half of the included articles (n = 23) were conducted in urban areas, while the remaining half (n = 23) were conducted in urban and rural mixed areas. Additionally, 34 (73.91%) articles had a sample size exceeding 10 000. Nearly half of the articles (n = 20, 43.48%) provided data on scoliosis prevalence in South Central China. The geographic distribution of all the included articles is shown in **Figure 2**. The detailed characteristics of all the included articles are listed in Table S5 in the **Online Supplementary Document**.

**Table 1:** Main characteristics of the included articles (n = 46)

Characteristics	IS (n = 45), n (%)	CS (n = 33), n (%)	Total (n = 46), n (%)
Year published			
*2000–09*	9 (20.00)	9 (27.27)	9 (19.56)
*2010–19*	30 (66.67)	22 (66.67)	30 (65.22)
*2020–23*	6 (13.33)	2 (6.06)	7 (15.22)
Setting			
*Urban*	23 (51.11)	17 (51.52)	23 (50.00)
*Rural*	0 (0.00)	0 (0.00)	0 (0.00)
*Mixed*	22 (48.88)	16 (48.48)	23 (50.00)
Sample size			
*≤1000*	2 (4.44)	0 (0.00)	2 (4.35)
*1001–5000*	3 (6.67)	1 (3.03)	3 (6.52)
*5001–10000*	7 (15.56)	5 (15.15)	7 (15.22)
*10001–20000*	16 (35.56)	12 (36.36)	16 (34.78)
*>20000*	17 (37.78)	15 (45.45)	18 (39.13)
Geographic region			
*North*	3 (6.67)	1 (3.03)	3 (6.52)
*South Central*	19 (42.22)	16 (48.48)	20 (43.48)
*East*	12 (26.67)	8 (24.24)	12 (26.08)
*Northeast*	3 (6.67)	3 (9.09)	3 (6.52)
*Northwest*	3 (6.67)	2 (6.06)	3 (6.52)
*Southwest*	5 (11.11)	3 (9.09)	5 (10.87)

CS – congenital scoliosis, IS – idiopathic scoliosis

### Estimated prevalence and cases of IS among Chinese children in 2020

**Figure 3:**
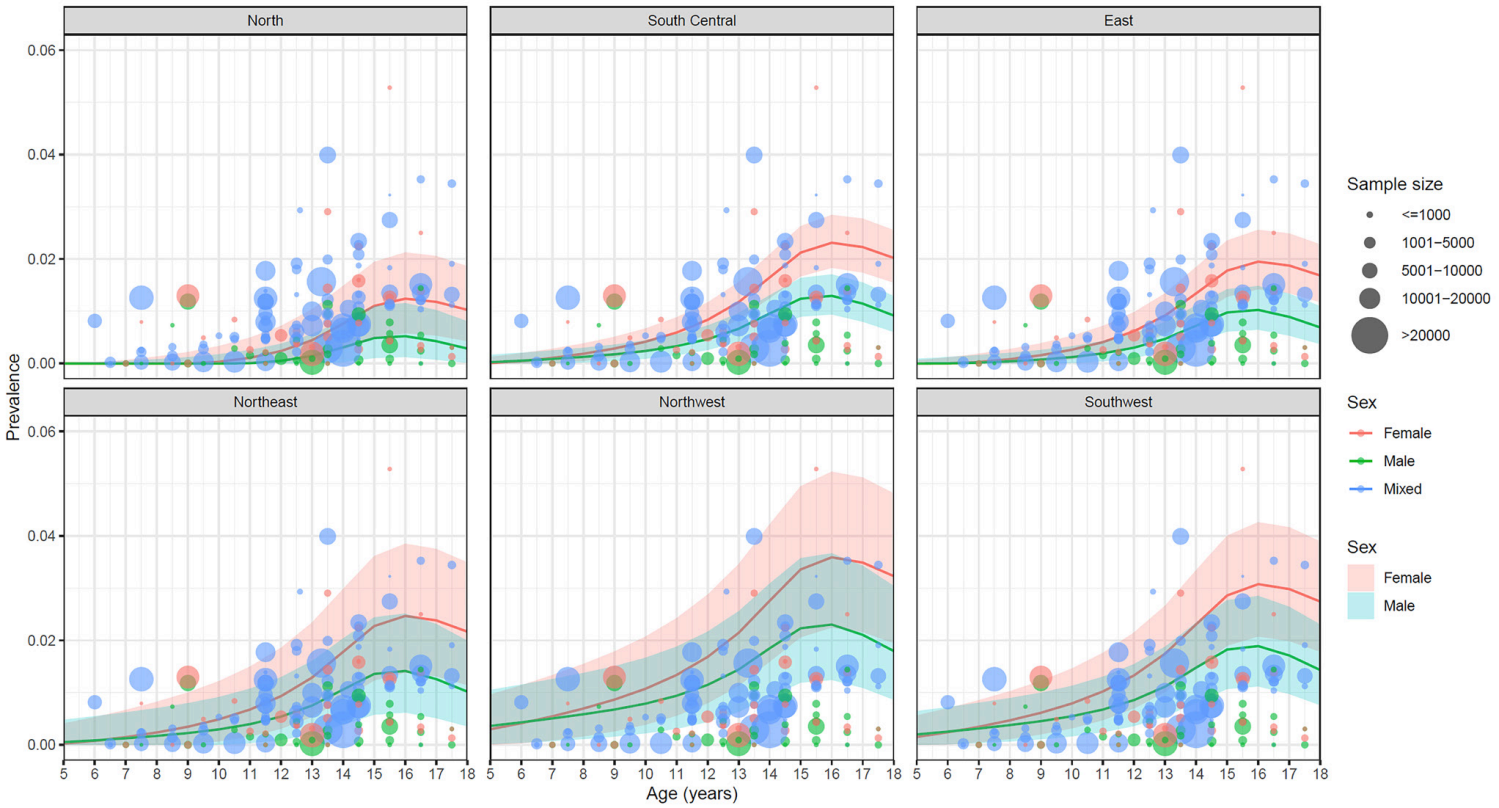
Estimated age- and sex-specific prevalence of IS in Chinese children, with 95% confidence interval. IS – idiopathic scoliosis

The age- and sex-specific prevalence of IS was estimated for six geographic regions in China (**Figure 3**, Table S6 in the **Online Supplementary Document**). Across all six geographic regions, the prevalence of IS peaked at the age of 16 years and then decreased, and it was consistently higher in girls than in boys.

**Table 2** shows the prevalence and the number of children with IS in China in 2020. In 2020, the overall prevalence of IS in Chinese Children aged 5–18 years was 0.79% (95% confidence interval (CI) = 0.45–1.27), translating to an estimated 1.81 million (95% CI = 1.04–2.91) children affected by IS. The prevalence of IS in boys increased from 0.07% (95% CI = 0.00–0.28) at age of five to 1.31% (95% CI = 0.80–1.97) at age of 16 and then decreased to 0.93% (95% CI = 0.50–1.50) at age 18, with a similar trend in girls (age five: 0.05%, 95% CI = 0.00–0.23; age 16: 2.32%, 95% CI = 1.62–3.17; age 18: 2.01%, 95% CI = 1.34–2.83). The overall prevalence of IS was nearly double in girls compared to boys (1.03%, 95% CI = 0.63–1.57 vs. 0.58%, 95% CI = 0.29–1.01), corresponding to 1.10 million (95% CI = 0.68–1.67) affected girls and 0.71 million (95% CI = 0.36–1.23) affected boys respectively.

**Table 2:** Age- and sex-specific prevalence and cases of scoliosis among Chinese children in 2020

	Prevalence, % (95% CI)			Cases*, n (95% CI)		
**Age in years**	**Boys**	**Girls**	**Overall**	**Boys**	**Girls**	**Overall**
5	0.07 (0.00–0.28)	0.05 (0.00–0.23)	0.06 (0.00–0.26)	5.79 (0.08–23.89)	3.74 (0.00–17.76)	9.53 (0.09–41.63)
6	0.09 (0.00–0.33)	0.09 (0.00–0.32)	0.09 (0.00–0.33)	8.48 (0.40–30.09)	7.03 (0.26–25.73)	15.52 (0.66–55.79)
7	0.13 (0.01–0.38)	0.15 (0.02–0.43)	0.14 (0.01–0.41)	11.01 (0.96–33.48)	11.28 (1.26–32.44)	22.29 (2.21–65.91)
8	0.16 (0.02–0.44)	0.23 (0.05–0.55)	0.19 (0.03–0.49)	15.20 (2.24–40.97)	17.98 (3.72–43.63)	33.17 (5.96–84.60)
9	0.21 (0.04–0.52)	0.32 (0.10–0.69)	0.26 (0.07–0.60)	18.77 (3.90–45.50)	24.61 (7.43–52.46)	43.38 (11.33–97.95)
10	0.28 (0.07–0.61)	0.45 (0.18–0.87)	0.36 (0.12–0.73)	24.21 (6.50–53.57)	34.13 (13.39–65.64)	58.35 (19.90–119.21)
11	0.37 (0.13–0.74)	0.63 (0.30–1.11)	0.49 (0.20–0.91)	31.64 (10.89–64.20)	47.07 (22.03–82.63)	78.69 (32.92–146.83)
12	0.50 (0.21–0.93)	0.87 (0.47–1.42)	0.67 (0.33–1.16)	44.10 (18.62–81.73)	66.74 (35.63–108.33)	110.84 (54.27–190.07)
13	0.70 (0.35–1.19)	1.21 (0.72–1.84)	0.94 (0.52–1.50)	59.51 (29.38–101.43)	89.87 (53.39–136.40)	149.37 (82.80–237.83)
14	0.99 (0.55–1.56)	1.69 (1.10–2.41)	1.32 (0.81–1.96)	83.07 (46.49–131.00)	123.10 (79.94–176.19)	206.18 (126.42–307.20)
15	1.27 (0.77–1.91)	2.15 (1.47–2.97)	1.68 (1.09–2.40)	108.97 (65.70–163.97)	161.09 (110.26–222.21)	270.07 (175.96–386.18)
16	1.31 (0.80–1.97)	2.32 (1.62–3.17)	1.79 (1.18–2.53)	115.33 (70.16–172.54)	179.69 (124.89–245.13)	295.01 (195.05–417.65)
17	1.16 (0.67–1.78)	2.23 (1.53–3.06)	1.66 (1.07–2.38)	102.24 (59.57–157.34)	174.54 (119.79–240.28)	276.79 (179.37–397.63)
18	0.93 (0.50–1.50)	2.01 (1.34–2.83)	1.44 (0.89–2.13)	83.26 (44.59–135.13)	160.65 (107.17–225.68)	243.91 (151.74–360.80)
Overall (5–18)	0.58 (0.29–1.01)	1.03 (0.63–1.57)	0.79 (0.45–1.27)	711.58 (359.48–1234.84)	1101.52 (679.16–1674.51)	1813.10 (1038.68–2909.28)

CI – confidence interval

*Cases are presented in thousand.

**Figure 4:**
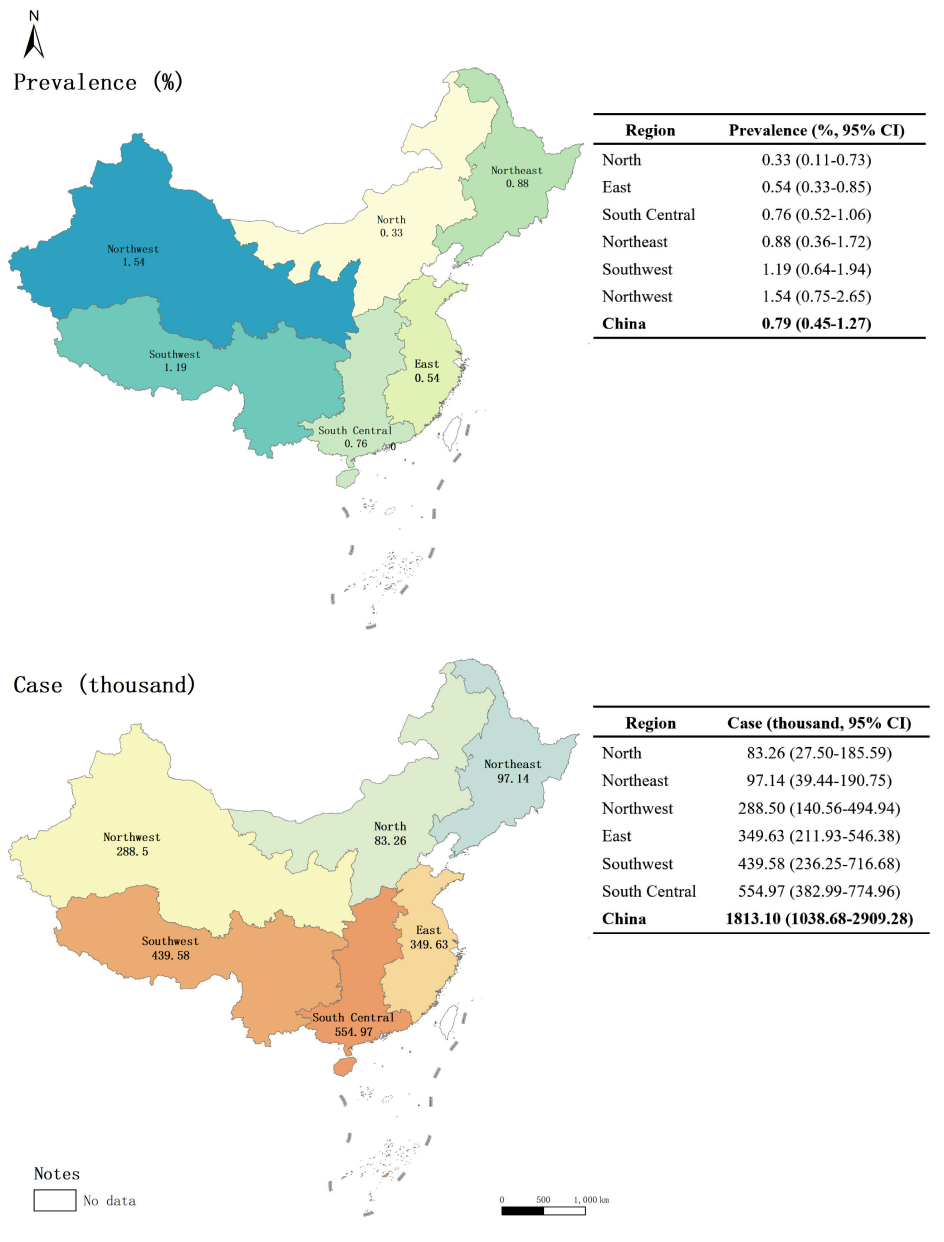
The regional prevalence and cases of IS among Chinese children aged 5–18 years in 2020. CI – confidence interval, IS – idiopathic scoliosis

As shown in **Figure 4**, the prevalence of IS varied across different geographic regions of China. In 2020, the prevalence of IS was the highest in Northwest China (1.54%, 95% CI = 0.75–2.65) and the lowest in North China (0.33%, 95% CI = 0.11–0.73). However, the largest number of children with IS resided in South Central China (554.97 thousand, 95% CI = 382.99–774.96), and the least resided in North China (83.26 thousand, 95% CI = 27.50–185.59).

### Pooled prevalence of IS with different curvatures and locations among Chinese children

**Figure 5:**
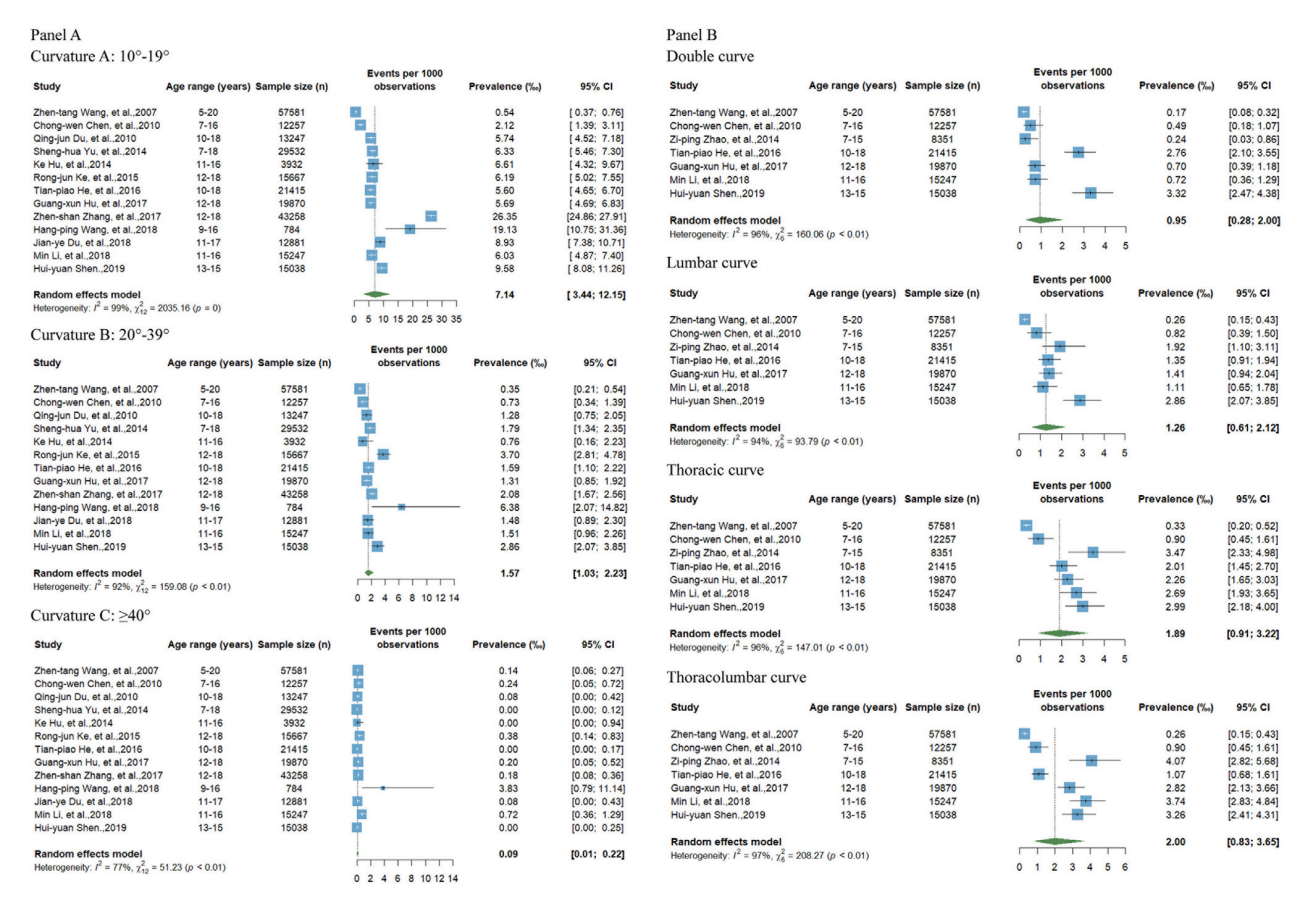
**Panel A**. Pooled prevalence of IS in different curvatures among Chinese children. **Panel B**. Pooled prevalence of IS in different curve locations among Chinese children. CI – confidence interval, IS – idiopathic scoliosis

**Figure 5****,** panel A presents the pooled prevalence of IS with different curvatures based on 13 articles. The prevalence of IS among Chinese children with curvatures of 10–19°, 20–39°, and ≥40° were 7.14‰ (95% CI = 3.44–12.15), 1.57‰ (95% CI = 1.03–2.33), and 0.09‰ (95% CI = 0.01–0.22), respectively. High heterogeneity was observed between articles in all four curvature groups (10–19°: *I^2^* = 99%, *P* = 0; 20–39°: *I^2^* = 92%, *P* < 0.01; ≥40°: *I^2^* = 77%, *P* < 0.01). According to the leave-one-out sensitivity analysis, no single article significantly affected the overall pooled prevalence in the meta-analyses (Figure S3, panel A in the **Online Supplementary Document**). As shown in Figure S4 in the **Online Supplementary Document**, no evidence for publication bias was found by funnel plot, Egger’s test (10–19°: *P* = 0.783; 20–39°: *P* = 0.191; ≥40°: *P* = 0.288) and Begg’s test (10–19°: *P* = 0.393; 20–39°: *P* = 0.542; ≥40°: *P* = 0.329).

**Figure 5****,** panel B presents the pooled prevalence of IS with different locations based on seven articles. The most common location of IS among Chinese children was the thoracolumbar curve, with a prevalence of 2.00‰ (95% CI = 0.83–3.65). High heterogeneity was observed between articles in four curve locations of IS (double curve: *I^2^* = 96%, *P* < 0.01; lumbar curve: *I^2^* = 94%, *P* < 0.01; thoracic curve: *I^2^* = 96%, *P* < 0.01; thoracolumbar curve: *I^2^* = 97%, *P* < 0.01. According to the leave-one-out sensitivity analysis, no single article has a significant effect on the overall pooled prevalence in the meta-analysis (Figure S3, panel B in the **Online Supplementary Document**).

### Pooled prevalence of CS among Chinese children

**Figure 6:**
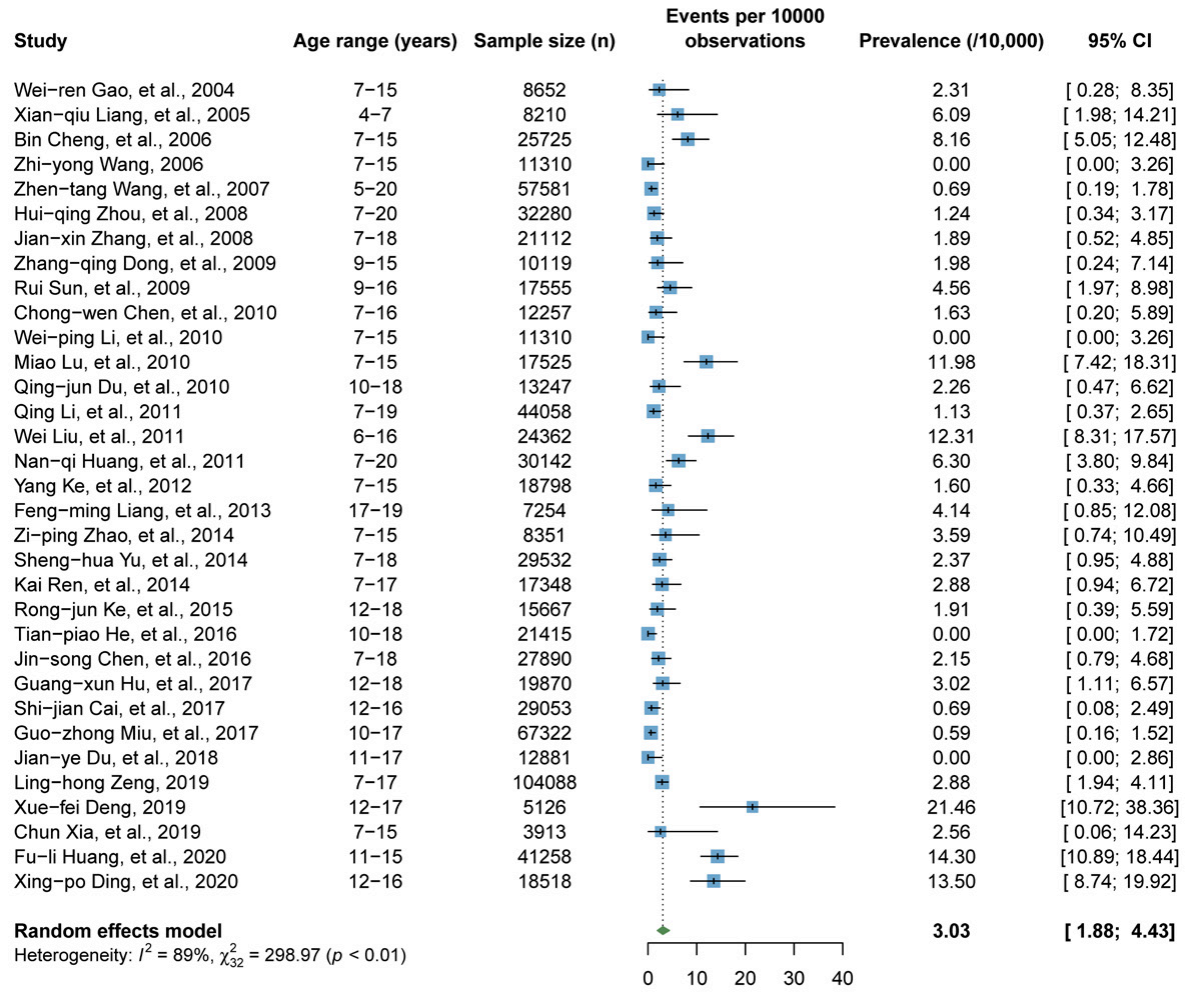
Pooled prevalence of CS among Chinese children. CI – confidence interval, CS – congenital scoliosis

**Figure 6** presents the pooled prevalence of CS based on 33 articles. The pooled prevalence of CS was 3.03 per 10 000 (95% CI = 1.88–4.43), with high heterogeneity observed between articles (*I ^2^* = 89%, *P* < 0.01). According to leave-one-out sensitivity analysis, the pooled prevalence of CS ranged from 2.76 per 10 000 (95% CI = 1.75–3.98) to 3.21 per 10 000 (95% CI = 2.02–4.65), with no significant effect on the overall estimated prevalence (Figure S5 in the **Online Supplementary Document****)**. As shown in Figure S6 in the **Online Supplementary Document**, no evidence for publication bias was found by funnel plot, Egger’s test (*P* = 0.346), and Begg’s test (*P* = 0.141).

## DISCUSSION

This systematic review and modelling study of 46 articles provides the most up-to-date evidence on the prevalence of scoliosis among Chinese children in mainland China. Our findings indicate that the prevalence of IS among Chinese children aged 5–18 years was 0.79% in 2020, with significant variations by age, sex, and geographic region. Specifically, IS was more common in children aged around 16 years than in other age groups and in girls than in boys. Across all six geographic regions, the prevalence of IS among children was the highest in Northwest China. Additionally, most children with scoliosis have a mild Cobb angle (10–19°) and a thoracolumbar curve. The prevalence of CS was relatively low, at 3.03 per 10 000 among Chinese children.

Our study found a marginally lower overall prevalence of IS among Chinese children than that reported in the previous systematic review and meta-analysis (0.79% vs. 0.93%) [28]. This discrepancy can be attributed to differences in data sources, paediatric population characteristics (e.g. age structure), and analysis methods utilised in the two studies. Specifically, compared to the previous study, our study was based on more than twice as many articles on the prevalence of IS among Chinese children, encompassing wider age ranges and larger sample sizes. Benefiting from our data extraction approach, we utilised multiple data points from the same article for our analyses, allowing us to estimate age- and sex-specific prevalence rates. Subsequently, our overall prevalence estimate for IS was adjusted to reflect the demographic structure in 2020.

As revealed in our study and previous studies, both boys and girls have a peak prevalence of IS at around 16 years of age, which can be attributed to the growth spurt phase [28,38]. The growth spurt occurs earlier in girls (at 11–13 years of age) than in boys (at 13–14 years of age), with a duration of around 3–4 years [39,40]. This may explain our finding of a higher prevalence of IS in girls than in boys, particularly after 10 years old, which is in line with previous research [18,28]. Another possible explanation for the sex difference in IS prevalence is leptin resistance in girls. The sudden increase in leptin during puberty can lead to the dysregulation of symmetrical spinal growth in girls [41]. Considering these findings, it is reasonable to consider setting different screening ages for boys and girls in screening programs.

Furthermore, our study found the highest prevalence of IS in Northwest China, which aligns with the findings of an epidemiological study published in 2022 in China [42], which revealed a higher prevalence of adolescent scoliosis in the western region (Gansu province) than in the eastern region (Shanghai province) [42]. This regional difference may be attributed to variations in socioeconomic development levels. Poor socioeconomic development is often associated with low health awareness and limited access to medical services, which may contribute to a higher prevalence of IS. Deficiencies in calcium, vitamin D, and other nutrients have also been confirmed as important factors affecting scoliosis [43]. Multiple studies have shown that children in Western China suffer from severe malnutrition and developmental delays [44,45]. Additionally, high altitude in Western China and the resulting lower oxygen concentration may lead to abnormal embryonic development, leading to defects in various organ systems [46]. However, our study did not confirm the association between prevalence and latitude, as reported in a previous systematic review [28]. Further multicentre and large-scale epidemiological surveys are still necessary to explore the relationship between IS prevalence and latitude.

Individuals with scoliosis having a curvature exceeding 20° require treatment and face a higher likelihood of undergoing surgery. Encouragingly, our study found that the prevalence of IS with a curvature between 10–19° was 7.14‰ among Chinese children, accounting for the highest proportion of all Chinese children with IS. This finding is in agreement with previous studies conducted worldwide. For instance, studies conducted in Korea, Tokyo, Turkey, and Norway reported that the majority of curvatures among children with IS fell within the range of 10–19° [18,47–49]. This prevalence can be attributed to high spinal plasticity during growth and development, early detection and monitoring, and the slower natural course and progression of mild scoliosis. In addition, the most common location of IS curves observed in our study was the thoracolumbar curve, which is consistent with studies from Singapore and Korea [13,50]. The thoracolumbar segment, situated between the thoracic and lumbar vertebrae, functions as a transitional area within the spine. It endures significant biomechanical stress, making it more susceptible to imbalances and lateral curvature of the spine [51]. Based on these findings, regular screening and monitoring are necessary for patients with a Cobb angle between 10–20° and a thoracolumbar curve.

Our study is the first to pool the prevalence of CS among Chinese children, with an estimate of 3.03 per 10 000. CS is an early-onset spinal deformity often accompanied by other systemic malformations affecting the spinal cord, heart, kidney, and gastrointestinal system [2]. The adverse health effects of CS, such as impaired lung function, are typically more severe than those associated with IS, and over 70% of CS cases progress aggressively, requiring surgical intervention [52]. Therefore, although the prevalence of CS in this study is relatively lower, the disease burden caused by CS should not be overlooked.

To the best of our knowledge, this study provides the most up-to-date and comprehensive estimate of scoliosis prevalence by age, sex, geographic region, and subtype in Chinese children, which can contribute to a more nuanced understanding of the epidemiology of scoliosis. Our systematic review employed rigorous and comprehensive search strategies to reduce selection bias, and a dual review process and rigorous selection criteria to minimise assessor bias. A multilevel mixed-effect meta-regression approach that incorporated many data points on IS prevalence made our estimated prevalence more robust. Moreover, this study only included studies conducted in the general Chinese children, which ensures the generalisability of our research findings.

However, our study has several limitations. First, the estimates of IS prevalence were derived using a meta-regression model that only incorporated age, sex, and geographic factors. Other potential confounders, such as study settings (urban or rural) and health behaviours, were not taken into account due to the absence of relevant data. Furthermore, due to the paucity of data in some provinces, our estimates of IS prevalence were generated at the regional level at best, which may limit the ability to reflect the true geographic variations of IS prevalence across China. Besides, Scoliosis is a curable condition. Our study only included cases identified through screening programs and did not count cases previously diagnosed but subsequently cured. This might have resulted in an underestimation of the overall prevalence, particularly in regions with superior treatment. Finally, for CS, the limited epidemiological evidence available restricted our ability to explore heterogeneity and bias by sub-group meta-analysis or meta-regression, resulting in only a crudely pooled estimate of prevalence.

The findings of this study significantly contribute to the existing scientific evidence on the epidemiology of scoliosis among Chinese children, with important implications for policymakers and health care providers. Despite the diagnostic criteria and methods for scoliosis being clear, and the Chinese government has shown increased attention to scoliosis in children, there remains a pressing need for national and local governments to develop appropriate screening strategies targeting different paediatric populations, especially girls, adolescents, and regions in northwest China. In the meantime, conducting more epidemiological surveys in regions with insufficient primary data are necessary to obtain robust epidemiological evidence of scoliosis. Moreover, further research is needed to identify the underlying mechanisms and risk factors associated with scoliosis in different age groups, sexes, regions, and subtypes, which can inform the development of effective prevention and management strategies for this condition.

## CONCLUSIONS

In conclusion, this comprehensive systematic review and modelling study sheds light on the prevalence of scoliosis among general Chinese children. Our results highlight considerable variation in scoliosis prevalence among different ages, sexes, and geographic regions. These findings may contribute to the development of targeted public health strategies by local governments and the optimisation of regional health resource distribution. Future high-quality epidemiological research is still needed to assess the variations of scoliosis burden in children.

## Additional material


Online Supplementary Document
