# Supplementary material for: Variations in the prevalence of scoliosis by age, sex, geographic region, and subtype among Chinese children: A systematic review and modelling study
Source: J Glob Health. 2024 Apr 12;14:04062. doi: 10.7189/jogh.14.04062 (PMC11009895; doi:10.7189/jogh.14.04062)
Supplement: Online Supplementary Document [file jogh-14-04062-s001.pdf]

Tables and figures legends

Table S1. Search strategies in bibliographic databases

Table S2. Quality for the included articles (n=46)

Table S3. Univariable and multilevel mixed-effect meta-regression models of various factors of IS

Notes: Age1-Age4 were variables generated in the process of restricted cubic spline, and the knots were 7.5,13.5,15,17.5,18.5; 95% CI, 95% confidence interval.

Table S4. Detailed description of estimated prevalence of IS

Table S5. Characteristics of the included articles (n=46)

Notes: IS, idiopathic scoliosis; CS, congenital scoliosis; NS, neuromuscular scoliosis; NA, not available.

Table S6. Estimated age- and sex-specific prevalence of IS in Chinese children, by geographic region (%, 95% CI)

Notes: 95% CI, 95% confidence interval.

Figure S1. The six geographic regions in China

Figure S2. Age range of data points of IS prevalence from included articles in Chinese children

Figure S3. Leave-one-out sensitivity analysis of the influence of single article on the pooled prevalence of IS in different curvatures (Panel A) and curve locations (Panel B) among Chinese children

Notes: IS, idiopathic scoliosis; 95% CI, 95% confidence interval.

Figure S4. Publication bias of the included articles of IS prevalence in different curvatures

Notes: IS, idiopathic scoliosis.

Figure S5. Leave-one-out sensitivity analysis of the influence of single article on the pooled prevalence of CS

Notes: CS, congenital scoliosis; 95% CI, 95% confidence interval.

Figure S6. Publication bias of the included articles of CS prevalence

Notes: CS, congenital scoliosis.

Table S1. Search strategies in bibliographic databases

Database	Access date	Subject category	Sub-database	Search terms	Publication date	Search method
CNKI	28/06/2023	Medicine & Public Health	Journal, Featured journal, Doctoral dissertation, Master dissertation	(SU % '青少年' + '少儿' + '学生' + '高中生' + '初中生' + '儿童' + '男孩' + '女孩' + '女生' + '男生' + '婴儿' + '幼儿' + '婴幼儿' + '新生儿') AND (SU % '脊柱侧弯' + '脊柱侧凸'+ '脊柱畸形' + '脊柱弯曲' + '脊柱侧突' + '龟背') AND (SU % '发病率' + '发生率' + '患病率' + '罹患率' + '现患率'+ '流行' + '现况' + '调查' + '监测')	01/01/1990-28/06/2023	Subject
Wanfang	28/06/2023	Medicine & Public Health	Journal articles, Dissertations	((((主题:"青少年" or 主题: "少儿" or 主题: "学生" or 主题: "高中生" or 主题: "初中生" or 主题: "儿童" or 主题: "男孩" or 主题: "女孩" or 主题: "男生" or 主题: "女生" or 主题: "婴幼儿" or 主题: "婴儿" or 主题: "幼儿" or 主题: "新生儿") AND (主题:"脊柱侧弯" or 主题: "脊柱侧凸" or 主题: "脊柱畸形" or 主题: "脊柱侧突" or 主题: "脊柱弯曲" or 主题: "龟背")) AND (主题:"发病率" or 主题: "发生率" or 主题: "患病率" or 主题: "罹患率" or 主题: "现患率" or 主题: "流行" or 主题: "现况" or 主题: "调查" or 主题: "监测")))	1990-2023	Subject (including title, keywords and abstract)
CBM	28/06/2023	Medicine & Public Health	All journals	((("青少年"[不加权:扩展] OR "儿童"[不加权:扩展] OR "学生"[不加权:扩展] AND "婴儿"[不加权:扩展] AND "婴儿，新生"[不加权:扩展] OR "青少年"[常用字段:智能] OR "少儿"[常用字段:智能] OR "学生"[常用字段:智能] OR "高中生"[常用字段:智能] OR "初中生"[常用字段:智能] OR "儿童"[常用字段:智能] OR "男孩"[常用字段:智能] OR "女孩"[常用字段:智能] OR "女生"[常用字段:智能] OR "男生"[常用字段:智能] OR "婴儿"[常用字段:智能] OR "婴幼儿"[常用字段:智能] OR "幼儿"[常用字段:智能] OR "新生儿"[常用字段:智能]) AND ("脊柱侧凸"[不加权:扩展] OR "脊柱弯曲"[不加权:扩展] OR"脊柱侧弯"[常用字段:智能] OR "脊柱侧凸"[常用字段:智能] OR "脊柱畸形"[常用字段:智能] OR "脊柱弯曲"[常用字段:智能] OR "脊柱侧突"[常用字段:智能] OR "龟背"[常用字段:智能])) AND ("发病率"[不加权:扩展] OR "患病率"[不加权:扩展] OR "发病率"[常用字段:智能] OR "患病率"[常用字段:智能] OR "现患率"[常用字段:智能] OR "罹患率"[常用字段:智能] OR "流行"[常用字段:智能] OR "现况"[常用字段:智能] OR "调查"[常用字段:智能] OR "监测"[常用字段:智能]))	1990-2023	Title, keywords, and abstract
CQVIP	28/06/2023	Medicine & Public Health	Not applicable	(M=(青少年 OR 少儿 OR 学生 OR 高中生 OR 初中生 OR 儿童 OR 男孩 OR 女孩 OR 女生 OR 男生 OR 婴儿 OR 婴幼儿 OR 幼儿 OR 新生儿) OR R=(青少年 OR 少儿 OR 学生 OR 高中生 OR 初中生 OR 儿童 OR 男孩 OR 女孩 OR 女生 OR 男生 OR 婴儿 OR 婴幼儿 OR 幼儿 OR 新生儿))	1990-2023	Title and keywords

Database	Access date	Subject category	Sub-database	Search terms	Publication date	Search method
				AND (M=(脊柱侧弯 OR 脊柱侧凸 OR 脊柱畸形 OR 脊柱弯曲 OR 脊柱侧突 OR 龟背) OR R=(脊柱侧弯 OR 脊柱侧凸 OR 脊柱畸形 OR 脊柱弯曲 OR 脊柱侧突 OR 龟背)) AND (M=(发病率 OR 发生率 OR 患病率 OR 现患率 OR 罹患率 OR 流行 OR 现况 OR 调查 OR 监测) OR R=(发病率 OR 发生率 OR 患病率 OR 现患率 OR 罹患率 OR 流行 OR 现况 OR 调查 OR 监测))		
PubMed	28/06/2023	Not applicable	Not applicable	(((((adolesc*[Title/Abstract] OR teen* [Title/Abstract] OR teenager*[Title/Abstract] OR young people[Title/Abstract] OR infant*[Title/Abstract] OR boys[Title/Abstract] OR girls[Title/Abstract] OR child*[Title/Abstract] OR youth* [Title/Abstract] OR pediatric*[Title/Abstract] OR paediatric*[Title/Abstract] OR toddler*[Title/Abstract] OR student*[Title/Abstract] OR juvenil*[Title/Abstract] OR newborn*[Title/Abstract])) AND ((scolio*[Title/Abstract] OR spinal disease*[Title/Abstract] OR spine disease*[Title/Abstract] OR spine deformation[Title/Abstract] OR spinal deformation[Title/Abstract] OR spinal curv*[Title/Abstract] OR spine curv*[Title/Abstract] OR spinal deformit*[Title/Abstract] OR spine deformit*[Title/Abstract] OR vertebral deformit*[Title/Abstract] OR vertebral anomal*[Title/Abstract]))) AND ((prevalen*[Title/Abstract] OR epidemiolog*[Title/Abstract] OR inciden*[Title/Abstract] OR morbidit* [Title/Abstract] OR attack rate[Title/Abstract] OR point prevalence[Title/Abstract]))) AND (((((China[Title/Abstract]) OR (Chinese[Title/Abstract])) OR (Hongkong[Title/Abstract])) OR (Macao[Title/Abstract])) OR (Taiwan[Title/Abstract]))) AND (("1990/01/01"[Date - Publication] : "3000"[Date - Publication]))	01/01/1990-28/06/2023	Title and abstract
EMBASE	28/06/2023	Not applicable	Not applicable	1 'adolescence'/exp OR 'adolescent'/exp OR 'young people'/exp OR 'infant'/exp OR 'boy'/exp OR 'girl'/exp OR 'child'/exp OR 'pediatric'/exp OR 'toddler'/exp OR 'student'/exp OR 'newborn'/exp OR 'juvenile'/exp OR 'adolesc*':ab,ti OR 'teen*':ab,ti OR 'teenager*':ab,ti OR 'young people':ab,ti OR 'infant*':ab,ti OR 'boys':ab,ti OR 'girls':ab,ti OR 'child*':ab,ti OR 'youth*':ab,ti OR 'pediatric*':ab,ti OR 'paediatric*':ab,ti OR 'toddler*':ab,ti OR 'juvenil*':ab,ti OR 'newborn*':ab,ti OR 'student*':ab,ti<br>2 'scoliosis'/exp OR 'spine disease'/exp OR 'spine malformation'/exp OR 'spinal curvature'/exp OR 'vertebra malformation'/exp OR 'scoliosis in infant and child'/exp OR 'idiopathic scoliosis'/exp OR 'scolio*':ab,ti OR 'spinal disease*':ab,ti OR 'spine disease*':ab,ti OR 'spine deformation':ab,ti OR 'spinal deformation':ab,ti OR 'spine malformation':ab,ti OR 'spinal malformation':ab,ti OR 'spinal curv*':ab,ti OR 'spine curv*':ab,ti OR 'spinal deformit*':ab,ti OR 'spine deformit*':ab,ti OR 'vertebral	1990-2023	Title and abstract

Database	Access date	Subject category	Sub-database	Search terms	Publication date	Search method
				deformit*':ab,ti OR 'vertebra malformation':ab,ti OR ' vertebral anomal*':ab,ti<br>3 'prevalence'/exp OR 'incidence'/exp OR 'epidemiology'/exp OR 'morbidity'/exp OR 'attack rate'/exp OR 'point prevalence'/exp OR 'prevalen*':ab,ti OR 'inciden*':ab,ti OR 'epidemiolog*':ab,ti OR 'morbidit*':ab,ti OR 'attack rate':ab,ti OR 'point prevalence':ab,ti<br>4 'china'/exp OR 'chinese'/exp OR 'hong kong'/exp OR 'macao'/exp OR 'taiwan'/exp OR 'china':ab,ti OR 'chinese':ab,ti OR 'hongkong':ab,ti OR 'macao':ab,ti OR 'taiwan':ab,ti<br>5 #1 AND #2 AND #3 AND #4<br>6 #1 AND #2 AND #3 AND #4 AND [1990-2022]/py<br>7 #6 AND [embase]/lim NOT ([embase]/lim AND [medline]/lim)		
Medline	28/06/2023	Not applicable	Not applicable	1 exp Infant/ or exp Child/ or exp Pediatrics/ or exp Students/or exp Adolescent/ or exp Infant, Newborn/ or (adolesc* or teen* or teenager* or young people or infant* or boys or girls or child* or youth* or pediatric* or paediatric* or toddler* or juvenil* or student* or newborn*).ab,ti.<br>2 exp Scoliosis/ or exp Spinal Diseases/ or (scolio* or spinal disease* or spine disease* or spine deformation or spinal deformation or spine malformation or spinal malformation or spinal curv* or spine curv* or spinal deformit* or spine deformit* or vertebral deformit* or vertebra malformation or vertebral anomal*).ab,ti.<br>3 exp Prevalence/ or exp Incidence/ or exp Epidemiology/ or exp Morbidity/ or (prevalen* or inciden* or epidemiolog* or morbidit* or attack rate or point prevalence).ab,ti.<br>4 exp China/ or exp Hong Kong/ or exp Macau/ or exp Taiwan/ or (China or Chinese or Hongkong or Macao or Taiwan).ab,ti.<br>5 1 and 2 and 3 and 4<br>6 limit 7 to (humans and yr="1990 -Current")	1990-2023	Title and abstract

Table S2. Quality assessment for the included articles (n=46)

ID	Author	Year published	Was the sample representative of the target population?	Were study participants recruited in an appropriate way?	Was the sample size adequate?	Were the study subjects and setting described in detail?	Is the data analysis conducted with sufficient coverage of the identified sample?	Were objective, standard criteria used for measurement of the condition?	Was the condition measured reliably?	Was there appropriate statistical analysis?	Are all important confounding factors/ subgroups/differences identified and accounted for?	Total scores
1	Qing Li, et al.[1]	2011	1	1	1	0	1	1	1	0	0	6
2	Zhentang Wang, et al.[2]	2007	1	1	1	1	1	1	1	1	1	9
3	Chong-wen Chen, et al.[3]	2010	1	1	1	1	1	1	1	1	1	9
4	Yu Zhang, et al.[4]	2016	1	1	1	1	0	1	0	1	1	7
5	Fu-li Huang, et al.[5]	2020	1	0	0	0	1	1	0	1	1	5
6	Miao Hu,et al.[6]	2022	1	0	0	1	0	1	1	1	1	6
7	Lei Zhu, et al.[7]	2021	1	0	0	1	1	1	0	0	0	4
8	Tao Xu, et al.[8]	2020	1	0	1	1	1	1	0	1	1	7
9	Xing-po Ding, et al.[9]	2020	1	1	1	1	1	1	1	1	1	9
10	Ling-hong Zeng[10]	2019	1	1	1	1	1	1	1	1	1	9
11	Xue-fei Deng, et al.[11]	2019	1	0	0	1	1	1	1	1	1	7
12	Hang-ping Wang ,et al.[12]	2018	1	0	1	0	1	1	1	1	1	7
13	Jian-ye Du, et al.[13]	2018	1	1	0	0	0	1	1	0	0	4
14	Min Li, et al.[14]	2018	1	0	0	1	1	1	1	1	1	7
15	Guang-xun Hu, et al.[15]	2017	1	1	0	1	1	1	1	1	1	8
16	Zhen-shan Zhang, et al.[16]	2017	1	1	1	1	1	1	1	1	1	9
17	Qian-ru Tang,et al.[17]	2017	1	0	0	0	0	1	0	1	1	4
18	Tian-piao He, et al.[18]	2016	1	0	0	1	1	1	1	0	0	5
19	Jin-song Chen, et al.[19]	2016	1	1	1	1	1	1	0	1	1	8
20	Rong-jun Ke, et al.[20]	2015	1	0	0	1	1	1	1	1	1	7
21	Jian-guo Ma, et al.[21]	2015	1	1	0	0	0	1	1	1	1	6
22	Zi-ping Zhao, et al.[22]	2014	1	0	0	1	1	1	1	1	1	7
23	Feng-ming Liang, et al.[23]	2013	1	0	0	0	1	1	0	1	1	5

ID	Author	Year published	Was the sample representative of the target population?	Were study participants recruited in an appropriate way?	Was the sample size adequate?	Were the study subjects and setting described in detail?	Is the data analysis conducted with sufficient coverage of the identified sample?	Were objective, standard criteria used for measurement of the condition?	Was the condition measured reliably?	Was there appropriate statistical analysis?	Are all important confounding factors/ subgroups/differences identified and accounted for?	Total scores
24	Yang Ke, et al.[24]	2012	1	1	1	1	1	1	1	1	1	9
25	Wei Liu, et al.[25]	2011	1	1	1	1	1	1	0	1	1	8
26	Wei-ping Li, et al.[26]	2010	1	0	0	0	1	1	1	1	1	6
27	Miao Lu, et al.[27]	2010	1	0	0	1	0	1	1	1	1	6
28	Qing-jun Du, et al.[28]	2010	1	1	0	0	1	1	1	1	1	7
29	Zhang-qing Dong, et al.[29]	2009	1	1	1	1	0	1	1	0	1	7
30	Rui Sun, et al.[30]	2009	1	1	1	1	0	1	1	0	1	7
31	Hui-qing Zhou, et al.[31]	2008	1	1	1	1	1	1	1	1	1	9
32	Jian-xin Zhang, et al.[32]	2008	1	1	0	1	1	1	1	1	1	8
33	Bin Cheng, et al.[33]	2006	1	1	1	1	0	1	1	1	1	8
34	Xian-qiu Liang, et al.[34]	2005	1	1	1	1	1	1	1	0	1	8
35	Sheng-hua Yu, et al.[35]	2014	1	1	1	1	1	1	1	1	1	9
36	Shi-jian Cai, et al.[36]	2017	1	1	1	1	1	1	1	1	1	9
37	Ting Hu, et al.[37]	2022	1	0	0	1	1	1	1	1	1	7
38	Zhi-yong Wang[38]	2006	1	1	0	1	1	1	1	1	1	8
39	Chun Xia, et al.[39]	2019	1	1	1	1	1	1	1	1	1	9
40	Hui-yuan Shen[40]	2019	1	0	0	1	0	1	1	1	1	6
41	Guo-zhong Miu, et al.[41]	2017	1	0	0	1	0	1	1	1	1	6
42	Wei-ren Gao, et al.[42]	2004	1	1	1	1	0	1	1	0	1	7
43	Kai Ren, et al[43]	2014	1	1	1	1	1	1	1	1	1	9
44	Nan-qi Huang, et al.[44]	2011	1	1	1	1	1	1	1	1	1	9
45	Ke Hu, et al.[45]	2014	1	0	0	1	1	1	1	0	1	6
46	Run Yang, et al.[46]	2022	1	0	0	1	1	1	1	1	1	7

Table S3. Univariable and multilevel mixed-effect meta-regression models of various factors of IS

Variable	β	No. of data points	*P*-value	95% CI
**Univariable meta-regression**				
Average age				
Age_1	0.0069	167	<0.0001	0.0049-0.0090
Age_2	0.0122	167	<0.0001	0.0078-0.0165
Age_3	-0.2402	167	<0.0001	-0.3278-0.1526
Age_4	0.3145	167	0.0012	0.1249-0.5042
Girl proportion	0.0266	167	<0.0001	0.0224-0.0308
Geographic region				
South Central	0.0387	167	0.0171	0.0069-0.0706
East	0.0255	167	0.1336	-0.0078-0.0588
Northeast	0.0422	167	0.0604	-0.0018-0.0862
Northwest	0.0468	167	0.0387	0.0024-0.0911
Southwest	0.0560	167	0.0051	0.0168-0.0951
Latitude	-0.0009	167	0.1751	-0.0022-0.0004
Longitude	-0.0011	167	0.1626	-0.0027-0.0004
Publish year	0.0010	167	0.2882	-0.0008-0.0028
Study year	0.0009	167	0.3133	-0.0009-0.0027
**Multilevel mixed-effect meta-regression**				
Intercept	-0.0386	167	0.0632	-0.0792-0.0021
Age_1	0.0051	167	<0.0001	0.0030-0.0073
Age_2	0.0121	167	<0.0001	0.0078-0.0165
Age_3	-0.2447	167	<0.0001	-0.3323--0.1571
Age_4	0.3267	167	0.0007	0.1370-0.5164
Girl proportion	-0.0247	167	0.0379	-0.0481--0.0014
South Central	0.0402	167	0.0349	0.0028-0.0777
East	0.0279	167	0.1614	-0.0112-0.0671
Northeast	0.0453	167	0.0860	-0.0064-0.0971
Northwest	0.0779	167	0.0034	0.0258-0.1300
Southwest	0.0637	167	0.0065	0.0178-0.1096
Girl proportion∗ average age	0.0039	167	<0.0001	0.0022-0.0057

Notes: Age_1-Age_4 were variables generated in the process of restricted cubic spline, and the knots were 7.5,13.5,15,17.5,18.5; 95% CI, 95% confidence interval.

Table S4. Detailed description of estimated prevalence of IS

The Freeman-Tukey double arcsine transformation is given by:

$$t = \sin^{-1}\sqrt{\frac{n}{N+1}} + \sin^{-1}\sqrt{\frac{n+1}{N+1}}$$

where n is the number of people with IS, N is the number of participants.

Then, the prevalence of IS was established as a function of age, girl proportion, geographic region, and girl proportion*age:

$$t = \alpha + \beta_1 \times Age_1 + \beta_2 \times Age_2 + \beta_3 \times Age_3 + \beta_4 \times Age_4 + \beta_5 \times Girl\ proportion + \beta_6 \times Geographic\ region + \beta_6 \times Girl\ proportion \times Average\ age + u_i$$

A random effect (u_i) was added into the model to control the effects of multiple data points from the same study.

Thus, the prevalence of IS was:

$$P=(sin\frac{\alpha+\beta_1\times Age_1+\beta_2\times Age_2+\beta_3\times Age_3+\beta_4\times Age_4+\beta_5\times Girl\ proportion+\beta_6\times Geographic\ region+\beta_6\times Girl\ proportion\times Average\ age+u_i}{2})^2$$

Where α is the intercept term, β is the coefficient, Age_1- Age_4= variables derived from the process of fitting restricted cubic spline (knots: 7.5,13.5,15,17.5,18.5), u_i= variance of the study-level random effect.

Table S5. Characteristics of the included articles (n=46)

ID	Author	Year published	Province	Geographic region	Study setting	Investigation date	Sampling strategy	Method of examination	Diagnostic criteria of scoliosis	Age range (years)	Tested sample	Cases of IS	Cases of CS	Cases of NS
1	Qing Li, et al.[1]	2011	Guangdong	South Central	Mixed	10/1997-10/2009	Census	Radiography	Cobb≥10°	7-19	44058	129	5	0
2	Zhentang Wang, et al.[2]	2007	Beijing	North	Mixed	3/2005-1/2006	Census	Radiography	Cobb≥10°	5-20	57581	59	4	1
3	Chong-wen Chen, et al.[3]	2010	Liaoning	Northeast	Mixed	5/2006-8/2009	Census	Radiography	Cobb≥10°	7-16	12257	38	2	1
4	Yu Zhang, et al.[4]	2016	Jiangsu	East	Urban	3/2014-6/2014	Census	Radiography	Cobb≥10°	6-13	11024	11	NA	NA
5	Fu-li Huang, et al.[5]	2020	Guangdong	South Central	Mixed	7/2015-12/2017	NA	Radiography	Cobb≥10°	11-15 (13.30±2.40)	41258	646	59	NA
6	Miao Hu,et al.[6]	2022	Shanghai	East	Urban	2019	NA	Radiography	Cobb≥10°	11-16	10731	214	NA	NA
7	Lei Zhu, et al.[7]	2021	Henan	South Central	Mixed	9/2020-12/2020	NA	Radiography	Cobb≥10°	6-19	146362	767	NA	NA
8	Tao Xu, et al.[8]	2020	Gansu	Northwest	Mixed	3/2018-3/2019	Stratified cluster sampling	Radiography	Cobb≥10°	7-15 (11.06±1.84)	2490	24	NA	NA
9	Xing-po Ding, et al.[9]	2020	Henan	South Central	Urban	9/2019-11/2019	Census	Radiography	Cobb≥10°	12-16	18518	207	25	2
10	Ling-hong Zeng[10]	2019	Guangdong	South Central	Mixed	9/2017-3/2019	Census	Radiography	Cobb≥10°	7-17	104088	NA	30	18
11	Xue-fei Deng, et al.[11]	2019	Sichuan	Southwest	Mixed	2/2018-10/2018	Multistage stratified cluster sampling	Radiography	Cobb≥10°	12-17	5126	107	11	0
12	Hang-ping Wang ,et al.[12]	2018	Yunnan	Southwest	Urban	5/2017-7/2017	Stratified random cluster sampling	Radiography	Cobb≥10°	9-16 (12.61±3.39)	784	23	NA	NA

ID	Author	Year published	Province	Geographic region	Study setting	Investigation date	Sampling strategy	Method of examination	Diagnostic criteria of scoliosis	Age range (years)	Tested sample	Cases of IS	Cases of CS	Cases of NS
13	Jian-ye Du, et al.[13]	2018	Guangdong	South Central	Urban	10/2013-1/2015	Convenience sampling	Radiography	Cobb≥10°	11-17 (14.17±0.12)	12881	136	0	0
14	Min Li, et al.[14]	2018	Guangdong	South Central	Mixed	3/2015-6/2017	NA	Radiography	Cobb≥10°	11-16	15247	126	NA	NA
15	Guang-xun Hu, et al.[15]	2017	Guangdong	South Central	Urban	9/2015-6/2017	Random sampling	Radiography	Cobb≥10°	12-18	19870	143	6	2
16	Zhen-shan Zhang, et al.[16]	2017	Guangdong	South Central	Mixed	3/2015-3/2016	Census	Radiography	Cobb≥10°	12-18	43258	1238	NA	NA
17	Qian-ru Tang, et al.[17]	2017	Shanghai	East	Urban	2/2015-11/2015	NA	Radiography	Cobb≥10°	11-13	5327	102	NA	NA
18	Tian-piao He, et al.[18]	2016	Fujian	East	Urban	5/2015	Random cluster sampling	Radiography	Cobb≥10°	10-18	21415	154	0	0
19	Jin-song Chen, et al.[19]	2016	Shaanxi	Northwest	Urban	9/2013-1/2014	Census	Radiography	Cobb≥10°	7-18	27890	351	6	3
20	Rong-jun Ke, et al.[20]	2015	Jiangsu	East	Mixed	9/2013	NA	Radiography	Cobb≥10°	12-18	15667	161	3	2
21	Jian-guo Ma, et al.[21]	2015	Hainan	South Central	Mixed	5/2012-4/2013	Random sampling	Radiography	Cobb≥10°	10-16	6952	88	NA	NA
22	Zi-ping Zhao, et al.[22]	2014	Guangdong	South Central	Urban	7/2011-1/2012	NA	Radiography	Cobb≥10°	7-15	8351	81	3	1
23	Feng-ming Liang, et al.[23]	2013	Guangdong	South Central	Urban	2013	Convenience sampling	Radiography	Cobb≥10°	17-19	7254	93	3	0
24	Yang Ke, et al.[24]	2012	Guangdong	South Central	Urban	6/2009-9/2011	Census	Radiography	Cobb≥10°	7-15	18798	150	3	3
25	Wei Liu, et al.[25]	2011	Heilongjiang	Northeast	Mixed	10/2005-10/2009	Census	Radiography	Cobb≥10°	6-16	24362	368	30	13
26	Wei-ping Li, et al.[26]	2010	Guangdong	South Central	Urban	1/1996-11/1997	Cluster sampling	Radiography	Cobb≥10°	7-15	33798	350	6	5

ID	Author	Year published	Province	Geographic region	Study setting	Investigation date	Sampling strategy	Method of examination	Diagnostic criteria of scoliosis	Age range (years)	Tested sample	Cases of IS	Cases of CS	Cases of NS
						2/2005-12/2005					11310	134	0	3
27	Miao Lu, et al.[27]	2010	Heilongjiang	Northeast	Mixed	4/2007-5/2009	NA	Radiography	Cobb≥10°	7-15	17525	311	21	NA
28	Qing-jun Du, et al.[28]	2010	Guangdong	South Central	Mixed	3/2007-12/2009	Random sampling	Radiography	Cobb≥10°	10-18	13247	94	3	1
29	Zhang-qing Dong, et al.[29]	2009	Jiangxi	East	Mixed	3/2007-1/2009	Census	Radiography	Cobb≥10°	9-15	10119	62	2	0
30	Rui Sun, et al.[30]	2009	Guizhou	Southwest	Urban	10/2007-12/2007	Census	Radiography	Cobb≥10°	9-16	17555	175	8	NA
31	Hui-qing Zhou, et al.[31]	2008	Fujian	East	Mixed	3/2007-5/2008	Census	Radiography	Cobb≥10°	7-20	32280	229	4	1
32	Jian-xin Zhang, et al.[32]	2008	Fujian	East	Urban	9/2002-3/2005	Cluster sampling	Radiography	Cobb≥10°	7-18	21112	153	4	1
33	Bin Cheng, et al.[33]	2006	Shaanxi	Northwest	Mixed	4/2002-5/2002 and 4/2003-5/2003	Census	Radiography	Cobb≥10°	7-15	25725	321	21	1
34	Xian-qiu Liang, et al.[34]	2005	Guangdong	South Central	Urban	1/2003-12/2004	Census	Radiography	Cobb≥10°	4-7	8210	67	5	0
35	Sheng-hua Yu, et al.[35]	2014	Guangdong	South Central	Urban	3/2012-6/2014	Census	Radiography	Cobb≥10°	7-18 (13.93±5.78)	29532	240	7	3
36	Shi-jian Cai, et al.[36]	2017	Guangdong	South Central	Urban	10/2013-10/2015	Census	Radiography	Cobb≥10°	12-16	29053	218	2	0
37	Ting Hu, et al.[37]	2022	Sichuan	Southwest	Urban	2019	NA	Radiography	Cobb≥10°	6-12	928	23	NA	NA
38	Zhi-yong	2006	Guangdong	South Central	Urban	2005	Random	Radiography	Cobb≥10°	7-15	11310	134	0	3

ID	Author	Year published	Province	Geographic region	Study setting	Investigation date	Sampling strategy	Method of examination	Diagnostic criteria of scoliosis	Age range (years)	Tested sample	Cases of IS	Cases of CS	Cases of NS
	Wang[38]						sampling							
39	Chun Xia, et al.[39]	2019	Shanghai	East	Urban	2/2016-9/2017	Census	Radiography	Cobb≥10°	7-15	3913	32	1	NA
40	Hui-yuan Shen[40]	2019	Tianjin	North	Urban	10/2017-5/2018	NA	Radiography	Cobb≥10°	13-15	15038	187	NA	NA
41	Guo-zhong Miu, et al.[41]	2017	Jiangsu	East	Mixed	9/2015-12/2015	NA	Radiography	Cobb≥10°	10-17	67322	195	4	1
42	Wei-ren Gao, et al.[42]	2004	Jiangsu	East	Mixed	6/2002-12/2003	Census	Radiography	Cobb≥10°	7-15	8652	63	2	0
43	Kai Ren, et al[43]	2014	Sichuan	Southwest	Mixed	2008-2012	Census	Radiography	Cobb≥10°	7-17	17348	89	5	NA
44	Nan-qi Huang, et al.[44]	2011	Guangdong	South Central	Urban	11/2007-7/2009	Census	Radiography	Cobb≥10°	7-20	30142	192	19	0
45	Ke Hu, et al.[45]	2014	Jiangsu	East	Mixed	5/2013-10/2013	NA	Radiography	Cobb≥10°	11-16	3931	29	NA	NA
46	Run Yang, et al.[46]	2022	Shanxi	North	Mixed	2019	Cluster sampling	Radiography	Cobb≥10°	6-15	22564	71	NA	NA

Notes: IS, idiopathic scoliosis; CS, congenital scoliosis; NS, neuromuscular scoliosis; NA, not available.

Table S6. Estimated age- and sex-specific prevalence of IS in Chinese children, by geographic region (%, 95%CI)

Age (years)	Boys						Girls					
	North	South Central	East	Northeast	Northwest	Southwest	North	South Central	East	Northeast	Northwest	Southwest
5	0.00	0.03	0.00	0.05	0.36	0.20	0.00	0.01	0.00	0.03	0.30	0.15
	(0.00-0.01)	(0.00-0.17)	(0.00-0.09)	(0.00-0.48)	(0.01-1.05)	(0.00-0.65)	(0.00-0.00)	(0.00-0.12)	(0.00-0.06)	(0.00-0.41)	(0.00-0.96)	(0.00-0.56)
6	0.00	0.05	0.00	0.09	0.43	0.25	0.00	0.05	0.00	0.08	0.41	0.24
	(0.00-0.03)	(0.00-0.21)	(0.00-0.12)	(0.00-0.55)	(0.04-1.15)	(0.01-0.72)	(0.00-0.02)	(0.00-0.20)	(0.00-0.12)	(0.00-0.53)	(0.03-1.14)	(0.01-0.70)
7	0.00	0.09	0.02	0.13	0.51	0.31	0.00	0.11	0.03	0.15	0.55	0.35
	(0.00-0.05)	(0.00-0.25)	(0.00-0.16)	(0.00-0.62)	(0.07-1.26)	(0.03-0.81)	(0.00-0.07)	(0.01-0.28)	(0.00-0.19)	(0.00-0.67)	(0.09-1.34)	(0.05-0.86)
8	0.00	0.13	0.04	0.17	0.59	0.38	0.00	0.19	0.08	0.24	0.70	0.47
	(0.00-0.08)	(0.02-0.30)	(0.00-0.2)	(0.00-0.71)	(0.11-1.38)	(0.07-0.90)	(0.00-0.13)	(0.05-0.39)	(0.00-0.28)	(0.00-0.83)	(0.16-1.55)	(0.11-1.03)
9	0.00	0.17	0.07	0.23	0.68	0.45	0.00	0.29	0.16	0.35	0.87	0.61
	(0.00-0.12)	(0.05-0.36)	(0.00-0.26)	(0.00-0.80)	(0.16-1.51)	(0.11-1.00)	(0.00-0.22)	(0.12-0.51)	(0.02-0.39)	(0.01-1.00)	(0.26-1.79)	(0.20-1.23)
10	0.00	0.24	0.12	0.30	0.79	0.55	0.03	0.42	0.26	0.49	1.07	0.79
	(0.00-0.18)	(0.09-0.45)	(0.01-0.33)	(0.00-0.92)	(0.22-1.67)	(0.16-1.13)	(0.00-0.34)	(0.22-0.67)	(0.08-0.53)	(0.07-1.22)	(0.39-2.07)	(0.32-1.46)
11	0.01	0.33	0.19	0.40	0.94	0.68	0.11	0.59	0.41	0.68	1.34	1.02
	(0.00-0.26)	(0.15-0.56)	(0.04-0.43)	(0.03-1.08)	(0.31-1.88)	(0.24-1.31)	(0.00-0.5)	(0.35-0.88)	(0.18-0.72)	(0.16-1.5.)	(0.56-2.43)	(0.47-1.76)
12	0.05	0.46	0.30	0.54	1.15	0.86	0.24	0.83	0.62	0.93	1.68	1.33
	(0.00-0.38)	(0.26-0.73)	(0.11-0.58)	(0.09-1.30)	(0.44-2.17)	(0.36-1.55)	(0.00-0.72)	(0.55-1.17)	(0.33-0.98)	(0.31-1.86)	(0.79-2.88)	(0.70-2.15)
13	0.14	0.66	0.47	0.75	1.44	1.12	0.44	1.17	0.92	1.29	2.14	1.75
	(0.00-0.56)	(0.42-0.96)	(0.22-0.80)	(0.20-1.61)	(0.63-2.56)	(0.54-1.88)	(0.08-1.04)	(0.84-1.56)	(0.57-1.35)	(0.53-2.34)	(1.13-3.46)	(1.01-2.67)
14	0.31	0.96	0.72	1.06	1.85	1.48	0.76	1.65	1.34	1.78	2.76	2.31
	(0.02-0.83)	(0.66-1.31)	(0.41-1.11)	(0.38-2.04)	(0.91-3.09)	(0.81-2.34)	(0.26-1.49)	(1.25-2.09)	(0.91-1.85)	(0.87-2.99)	(1.59-4.22)	(1.45-3.35)
15	0.49	1.24	0.98	1.36	2.23	1.83	1.10	2.12	1.78	2.27	3.36	2.86
	(0.10-1.10)	(0.89-1.64)	(0.61-1.42)	(0.58-2.44)	(1.19-3.58)	(1.07-2.77)	(0.49-1.95)	(1.67-2.63)	(1.28-2.36)	(1.23-3.61)	(2.06-4.95)	(1.90-4.01)
16	0.52	1.30	1.03	1.42	2.30	1.89	1.24	2.31	1.95	2.47	3.59	3.08

Age (years)	Boys						Girls					
	North	South Central	East	Northeast	Northwest	Southwest	North	South Central	East	Northeast	Northwest	Southwest
	(0.12-1.16)	(0.94-1.71)	(0.65-1.48)	(0.62-2.52)	(1.24-3.67)	(1.12-2.85)	(0.58-2.13)	(1.83-2.85)	(1.42-2.56)	(1.38-3.85)	(2.24-5.23)	(2.07-4.26)
17	0.43	1.15	0.89	1.26	2.11	1.71	1.18	2.23	1.87	2.38	3.49	2.98
	(0.07-1.02)	(0.80-1.55)	(0.53-1.34)	(0.51-2.31)	(1.09-3.43)	(0.97-2.64)	(0.53-2.06)	(1.74-2.77)	(1.34-2.49)	(1.31-3.75)	(2.16-5.12)	(1.99-4.17)
18	0.29	0.92	0.69	1.02	1.80	1.43	1.03	2.02	1.68	2.17	3.23	2.74
	(0.01-0.81)	(0.60-1.29)	(0.37-1.10)	(0.35-2.00)	(0.86-3.04)	(0.76-2.30)	(0.43-1.86)	(1.55-2.55)	(1.17-2.28)	(1.14-3.49)	(1.95-4.81)	(1.78-3.89)

Notes: 95% CI, 95% confidence interval.

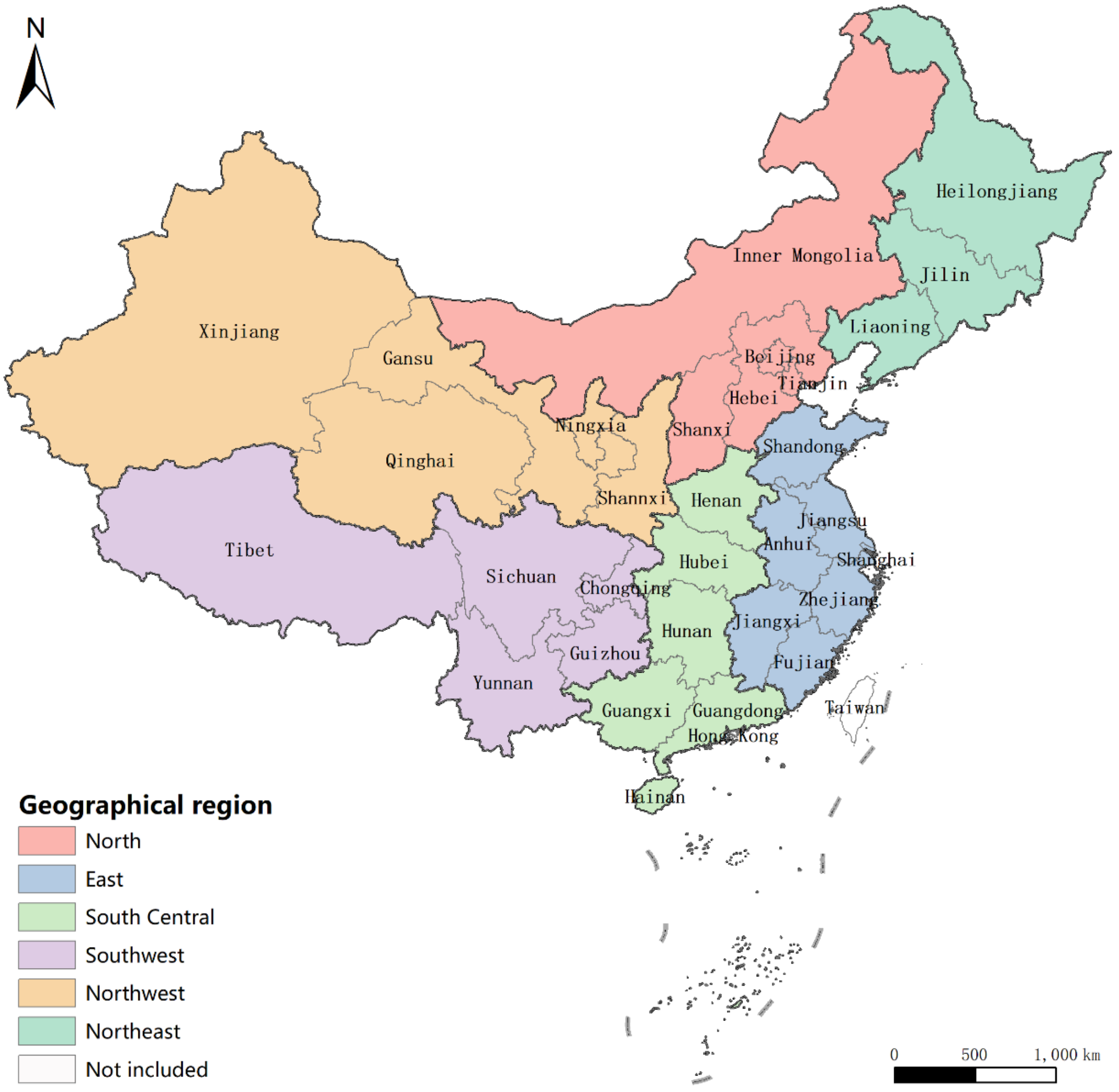

Figure S1. The six geographic regions in China

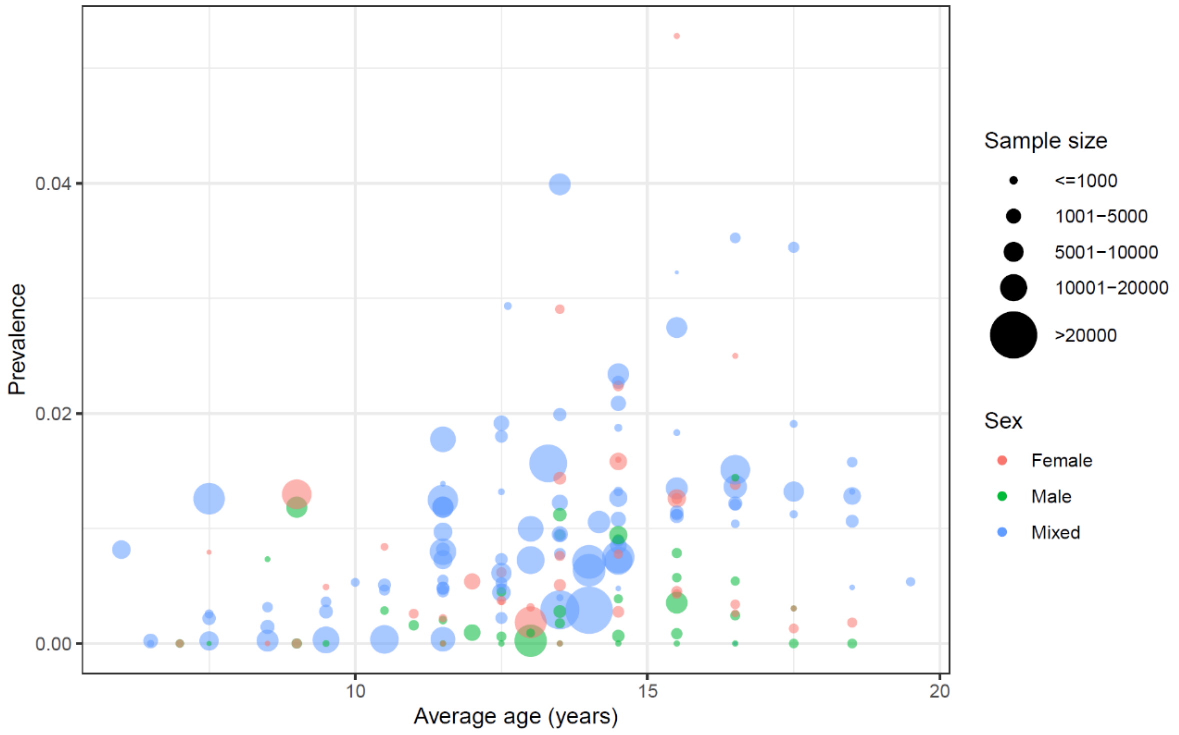

Figure S2. Age range of data points of IS prevalence from included articles in Chinese children

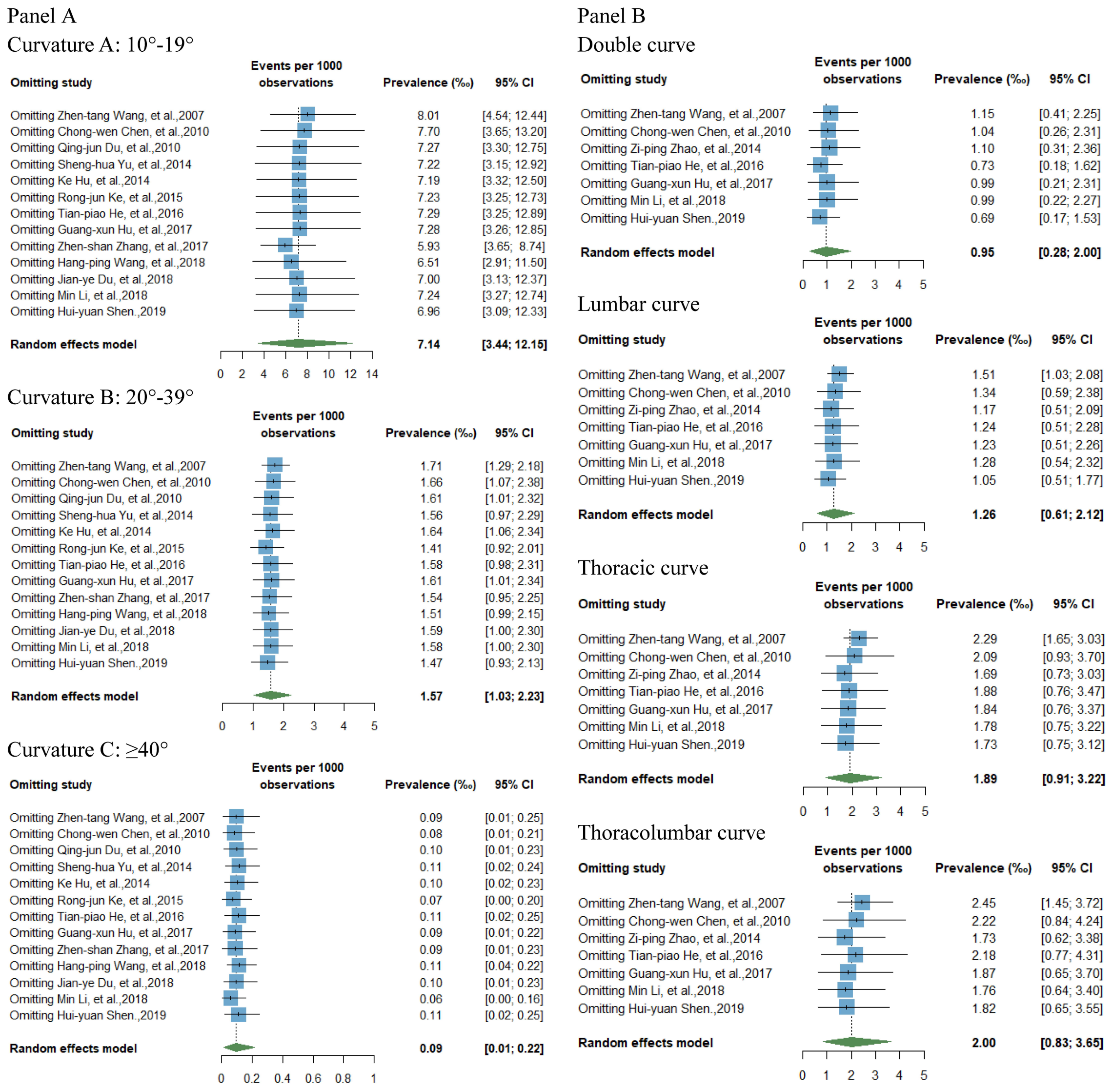

Figure S3. Leave-one-out sensitivity analysis of the influence of single article on the pooled prevalence of IS in different curvatures (Panel A) and curve locations (Panel B) among Chinese children

Notes: IS, idiopathic scoliosis; 95% CI, 95% confidence interval.

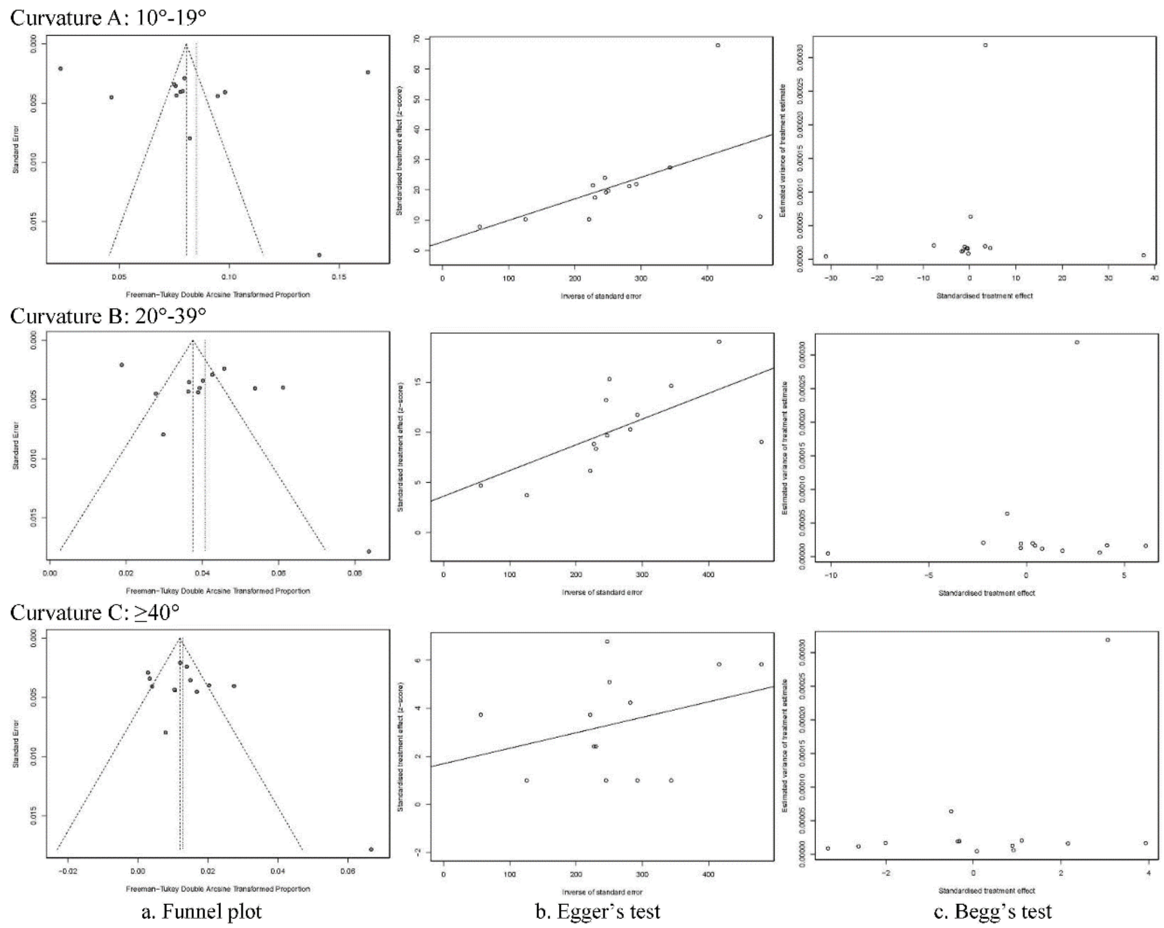

Figure S4. Publication bias of the included articles of IS prevalence in different curvatures among Chinese children

Notes: IS, idiopathic scoliosis.

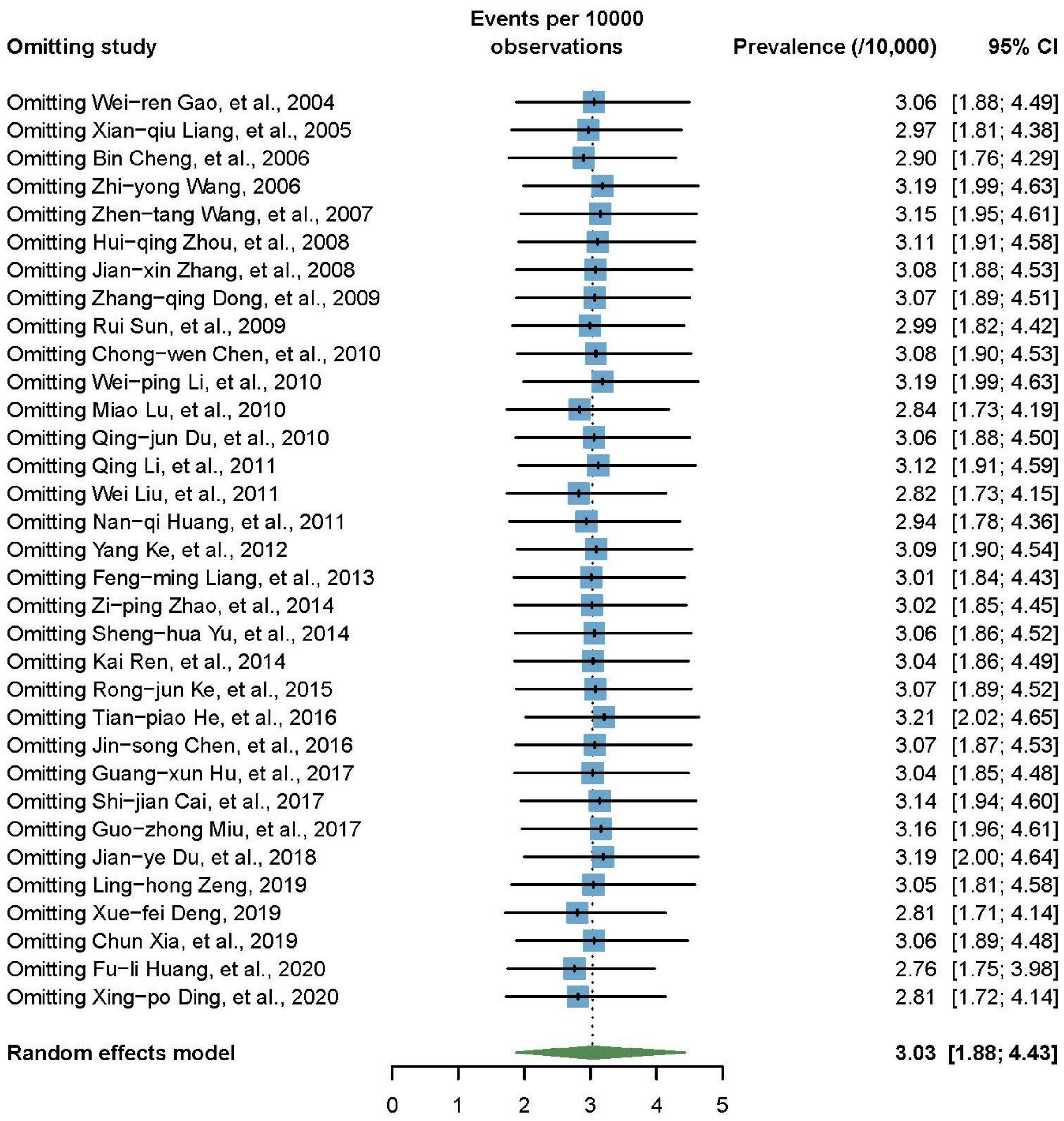

Figure S5. Leave-one-out sensitivity analysis of the influence of single article on the pooled prevalence of CS among Chinese children

Notes: CS, congenital scoliosis; 95% CI, 95% confidence interval.

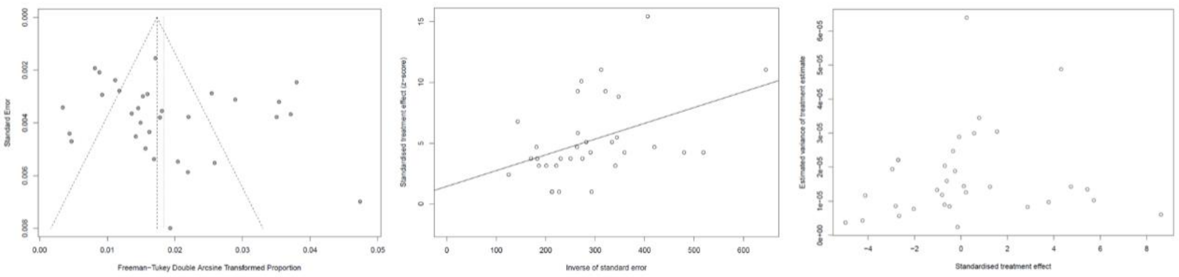

A. Funnel plot B. Egger's test C. Begg's test

Figure S6. Publication bias of the included articles of CS prevalence among Chinese children

Notes: CS, congenital scoliosis.

Reference

1. Qing Li, Yuan-xing Yuan, Liang D-c, Aiming Zhang, Zhi Mei, Chengyi Zhao. A survey on adolescent scoliosis under the step intervention. *Journal of Clinical Orthopaedics* 2011; **14**(5): 481-3.

2. Zhengtang W, Zhongshi L, Chaohui D, et al. Investigation of scoliosis among school children in Beijing. *Chinese Journal of Spine and Spinal Cord* 2007; **17**(6): 440-2.

3. Chong wen C, Bin T, Yi C, et al. Investigation of scoliosis among school children in Jinzhou of Liaoning. *Medical Innovation of China* 2010; **7**(8): 44-6.

4. Yu Z, Xiaojun W, Sun N. Prevalence of Idiopathic Scoliosis in Primary School Children in Beitang District, Wuxi, Jiangsu, China. *Chin J Rehabil Theory Pract* 2016; **22**(03): 335-40.

5. Huang F, Liu Y, Wu J, et al. Incidence of scoliosis among junior high school students in Zhongshan city, Guangdong and the possible importance of decreased miR-30e expression. *J Int Med Res* 2020; **48**(6): 300060519889438.

6. Hu M, Zhang Z, Zhou X, et al. Prevalence and determinants of adolescent idiopathic scoliosis from school screening in Huangpu district, Shanghai, China. *Am J Transl Res* 2022; **14**(6): 4132-8.

7. Lei Z, Weifeng Z, Yubin W. Investigation and rehabilitation intervention of primary and secondar school students with scoliosis in Zhengzhou in 2020. *J Medical Forum* 2021; **42**(12): 79-84.

8. Tao X, Jiancheng X, Zhonghua L, Jing J. Idiopathic scoliosis in primary and middle school students in Lanzhou. *Prev Med* 2020; **32**(11): 1155-7+60.

9. Xingpo D, Junyan T, Shuang C, et al. A survey of prevalence rate of idiopathic scoliosis of middle school students in Zhengdong new district of Zhengzhou. *J Trad Chin Orthop Trauma* 2020; **32**(06): 31-4+7.

10. Linghong Z. Prevalence of scoliosis among adolescents in Huizhou. *Shenzhen Journal of Integrated Traditional Chinese and Western Medicine* 2019; **29**(16): 196-7.

11. Xuefei D, Yulin W, Mingqiong D. Investigation on Current Situation of Adolescent Scoliosis in Ganzi Tibetan Autonomous Prefecture in 2018. *J Prev Med Inf* 2019; **35**(07): 667-70.

12. Hangping W, Zhenwu S, Tinghua W, Yimei D. Prevalence and risk factors of adolescent idiopathic scoliosis in Kunming. *Chin J Sch Health* 2018; **39**(12): 1851-4.

13. Jianye D, Shijian C, Biao J, Zijian Z, Zhifa M. Survey analysis of idiopathic in 12881 junior middle school students from Shantou city, Guangdong province. *Chin J Prim Med Pharm* 2018; **25**(15): 1976-9.

14. Min L, Li S, Hua Z, et al. An Investigation about the Prevalence Rate of Idiopathic Scoliosis in Middle School Students in Shenzhen. *Shenzhen Journal of Integrated Traditional Chinese and Western Medicine January* 2018; **28**(02): 3-5.

15. Guangxun H, Cheng L, He L, et al. Prevalence and prevention of idiopathic scoliosis among middle school students in Nanshan district of Shenzhen city. *CHINESE COMMUNITY DOCTORS* 2017; **33**(35): 110-1.

16. Zhenshan Z, Fuli H, Junzhe W, et al. Prevalence of idiopathic scoliosis among junior middle school students in Zhongshan. *Inner Mongolia Med J* 2017; **49**(05): 541-3.

17. Qianru T, Mingli Z, Yi S, Jing Z, Xiaoyan Y, Yawen G. Prevalence of idiopathic scoliosis among junior middle school students in Jing'an District, Shanghai. *Int J Orthop* 2017; **38**(03): 205-6.

18. Tianpiao H, Jingsong Z. Analysis the scoliosis in Jinjiang primary and middle school students. *The Medical Forum* 2016; **20**(36): 5081-2.

19. Jinsong C, Feng Y, Hongbin G, et al. Investigation of scoliosis among urban adolescents in Xi'an. *Shaanxi Medical Journal* 2016; **45**(03): 371-3.

20. Rongjun K, Xingbing C, Yonghui H, Fanggang L, Yan C, Fei L. Prevalence of adolescent idiopathic scoliosis of Zhenjiang. *Jiangsu Med J* 2015; **41**(18): 2130-2.

21. Jianguo M, Qiang L, Zhengwei C, Mingzhuo L, Zhiming G. Epidemiology of adolescent idiopathic scoliosis in Sanya. *Hainan Med J* 2015; **26**(13): 2000-2.

22. Ziping Z, Haifeng L, Zhiguo W, Jianzhong F, Dongdong W. Investigation and study of prevalence rate of adolescent scoliosis in Guangzhou Liwan district. *CHINA MODERN MEDICINE* 2014; **21**(19): 137-9.

23. Fengming L, Ruibing L. Analysis of 96 Cases of Scoliosis Found by DR Chest Photos in College Entrance Examination. *Clinical Medicine & Engineering* 2013; **20**(06): 773-4.

24. Yang K, Jiaxiong H, Zhixiong P. Prevalence of adolescent scoliosis in Foshan. *The Journal of Practical Medicine* 2012; **28**(05): 832-4.

25. Wei L, Qinghe C, Lun W, et al. A general survey of the adolescent scoliosis in Harbin and an analysis of the result of the brace treatment. *Orthopedic Journal of China* 2011; **19**(15): 1244-7.

26. Weiping L, Zhiyong W, Bin S, Shangli L, Huiyong S, Jianrong H. Scoliosis prevalence survey of adolescents in Guangzhou City during different periods. *Journal of Clinical Rehabilitative Tissue Engineering Research* 2010; **14**(46): 8712-6.

27. Lu M, Qinghe C, Jichang G, Chengmin Z, Jinliang Q. A general survey of adolescent scoliosis in Heilongjiang province. *Orthopedic Journal of China* 2010; **18**(07): 591-3.

28. Qingjun D, Haidong Y, Mingguang H, et al. A survey on the incidence of idiopathic scoliosis for primary and secondary school students in Shunde District. *Lingnan Modern Clinics in Surgery* 2010; **10**(01): 52-4.

29. Zhangqing D, Long X, Jiangping Z, Xiaozheng T, Qiang X. Investigation of Scoliosis Among School Children in Nanchang. *Acta Academiae Medicinae Jiangxi* 2009; **49**(02): 129-32.

30. Rui S, Xiaoyan Y, Guoqing L, et al. Analysis of the Prevalence of Scoliosis among Adolescents in the Central Urban Area of Liupanshui of Guizhou in 2007. *Guizhou Medical Journal* 2009; **33**(01): 73-4.

31. Huiqing Z, Jianxin Z, Sishun L. Epidemiological investigation of scoliosis among adolescents in Hui′ an county, Fujian province. *Chinese Journa Journal of Spine and Spinal Cord* 2008; (11): 824-7.

32. Jianxin Z, Guowen L, Xiaodong C, Tianhai G, Xianjun L. A Survey on the Incidence of Juvenile Scoliosis in Quanzhou Area. *Chinese J Trad Med Traum ＆ Orthop* 2008; (04): 1-4.

33. Bin C, Fengtao L, Jinhui S. A general survey and treatment of children and adolescent scoliosis in Xi′an. *Chinese Journal of Spine and Spinal Cord* 2006; (03): 180-2.

34. Xianqiu L, Shaoxian H, Bin Y. General survey and prevention of scoliosis in children in Zhaoqing of Guangdong. *Maternal and Child Health Care of China* 2005; (12): 1496-7.

35. Shenghua Y, Hansheng HU, Zhenbo FAN, Qinye QIU, Yuanhui LI. An Investigation on the Prevalence Rate of Idiopathic Scoliosis for Primary and Secondary School Students in Guangzhou. *Clinical Medicine & Engineering* 2014; (10): 1359-60.

36. Shijian C, Jianye D, Zijian Z, Jie W, Zhigang Z. Prevalence of scoliosis among junior middle school students in Shantou of Guangdong. *The World Clinical Medicine* 2017; **11**(16): 230,2.

37. Ting H, Jin-song Z, Zhuo-hui W, et al. Current situation and related factors of idiopathic scoliosis in primary school children from Qingyang district Chengdu. *Chin J Child Health Care* 2022; **30**(6): 617-21.

38. Zhiyong W. An Investigation of Scoliosis Case Rates among Adolescents of Guangzhou at Different Periods and Orthosis Treatment for Scoliosis: Sun Yat-sen University; 2006.

39. Chun X, Jinfa G, Ling M, Yaoyao C, Jie S. Investigation of prevalence of scoliosis among adolescents in a community of Jiading. *Shanghai Medical & Pharmaceutical Journal* 2019; **40**(08): 53-5.

40. Huiyuan S. Epidemiological survey of scoliosis among eighth grade students in six districts of Tianjin: Tianjin University of Sport; 2019.

41. Guozhong M, Chao X. Epidemiological investigation of scoliosis among adolescents in Jiangyin. *Jiangsu J Prev Med* 2017; **28**(02): 195-6+213.

42. Weiren G, Xiangyang N, Yunwei S. The application and revelation of "point Line" in scoliosis investigation. *HEBEI MEDICINE* 2004; **10**(12): 1073-5.

43. Kai R, Xiaoming G, Rong Z, Xiangbin C, Junjie Z, Jia L. Investigation of AIS among School Children in Zigong. *Sichuan Medical Journa* 2014; **35**(07): 853-5.

44. Nanqi H, Hushan G, Jin L, et al. A survey on adolescent scoliosis in Guangzhou. *Chin J Epidemiol* 2011; (02): 138-41.

45. Ke H, Rongfan X. A study on treating back diseases by moire pattern therapy. *Clinical Journal of Chinese Medicine* 2014; (3): 230-2.

46. Run Y, Yan R, Kecheng C, Zhigui L, Xianghui C, Weiji L. Study on the current status of spinal morphology and factors influencing it in primary and secondary school students in Taiyuan. *Journal of Shanxi University of Chinese Medicine* 2022; **23**(05): 452-5.
